# Ultra-Efficient and Cost-Effective Platinum Nanomembrane Electrocatalyst for Sustainable Hydrogen Production

**DOI:** 10.1007/s40820-024-01324-5

**Published:** 2024-02-05

**Authors:** Xiang Gao, Shicheng Dai, Yun Teng, Qing Wang, Zhibo Zhang, Ziyin Yang, Minhyuk Park, Hang Wang, Zhe Jia, Yunjiang Wang, Yong Yang

**Affiliations:** 1 Department of Mechanical Engineering, College of Engineering, City University of Hong Kong, Tat Chee Avenue, Kowloon Tong, Kowloon, Hong Kong People’s Republic of China; 2 State Key Laboratory of Nonlinear Mechanics, Institute of Mechanics, Chinese Academy of Sciences, Beijing, People’s Republic of China; 3 School of Engineering Science, University of Chinese Academy of Sciences, Beijing, People’s Republic of China; 4 Laboratory for Microstructures, Institute of Materials, Shanghai University, Shanghai, People’s Republic of China; 5 School of Materials Science and Engineering, Jiangsu Key Laboratory for Advanced Metallic Materials, Southeast University, Nanjing, People’s Republic of China; 6 Department of Materials Science and Engineering, College of Engineering, City University of Hong Kong, Tat Chee Avenue, Kowloon Tong, Kowloon, Hong Kong People’s Republic of China

**Keywords:** Platinum, Hydrogen evolution reaction, Lattice distortion, Heterogeneous strain

## Abstract

Hydrogen production through hydrogen evolution reaction (HER) offers a promising solution to combat climate change by replacing fossil fuels with clean energy sources. However, the widespread adoption of efficient electrocatalysts, such as platinum (Pt), has been hindered by their high cost. In this study, we developed an easy-to-implement method to create ultrathin Pt nanomembranes, which catalyze HER at a cost significantly lower than commercial Pt/C and comparable to non-noble metal electrocatalysts. These Pt nanomembranes consist of highly distorted Pt nanocrystals and exhibit a heterogeneous elastic strain field, a characteristic rarely seen in conventional crystals. This unique feature results in significantly higher electrocatalytic efficiency than various forms of Pt electrocatalysts, including Pt/C, Pt foils, and numerous Pt single-atom or single-cluster catalysts. Our research offers a promising approach to develop highly efficient and cost-effective low-dimensional electrocatalysts for sustainable hydrogen production, potentially addressing the challenges posed by the climate crisis.

**Supplementary Information:**

The online version contains supplementary material available at 10.1007/s40820-024-01324-5.

## Highlights

- A percolating network of distorted 2D Pt nanomembranes was synthesized by polymer surface buckling-enabled exfoliation for hydrogen evolution reaction.
- The 2D Pt nanomembrane enabled important technological applications for its high efficiency, low costs, and good stability, making it potential alternative to commercial Pt/C.
- Our 2D Pt nanomembranes offer insights into a new mechanism for efficient catalyst design strategy: lattice distortion-induced heterogeneous strain.

**Supplementary Information:**

The online version contains supplementary material available at 10.1007/s40820-024-01324-5.

## Graphical abstract

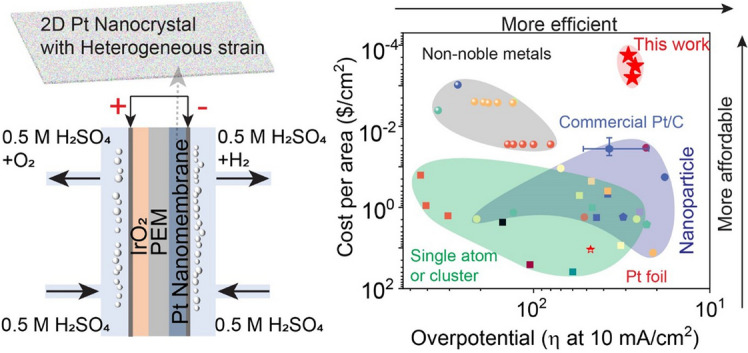

## Introduction

Dwindling fossil fuel supplies and environmental damage have led to extensive research on producing clean, sustainable hydrogen [1–3]. Electrochemical acidic water splitting is a promising hydrogen production method that offers benefits like fast kinetics and high hydrogen purity [4, 5]. Due to its effective binding energy, platinum (Pt) is one of the best catalysts for hydrogen evolution reaction (HER) with excellent features, such as low overpotential and high current density [6]. However, Pt’s scarcity and high costs limit its widespread application [7–9]. Consequently, there has been significant research interest in developing low-loading Pt-based catalysts that balance cost and efficiency. Despite remaining a popular research area for decades [7–17], the complex synthesis process and high cost associated with these catalysts have thus far prevented large-scale practical applications.

To date, various synthetic strategies have been developed to maximize the catalytic potential of Pt, such as wet chemical approaches [9–13, 18–22] and atomic layer deposition (ALD) [14–17]. For instance, Tiwari et al. [10] synthesized a multicomponent catalyst with an ultralow Pt loading, which was supported on melamine-derived graphitic tubes, following a multi-step process that involved grinding, 750 °C heating, acid leaching, and electrochemical deposition. In another example, Liu et al. [15] fabricated atomically dispersed Pt supported on curved carbon by oxidizing detonation nanodiamonds (DND) powders for 24 h at 160 °C, thermal deoxygenation, 4 h’ oxidation by HNO_3_, and finally depositing Pt via ALD. Although these Experimental Section have demonstrated promising results, they typically require high-temperature environments and/or expensive equipment, resulting in high energy consumption and high costs. Moreover, the yield rate of production is relatively low for these Experimental Section [23], making it vital to develop a cost-effective catalyst with a high yield rate for sustainable hydrogen production.

In this study, we employed the method of polymer surface buckling-enabled exfoliation (PSBEE) [24–26] to synthesize large area, freestanding 2D Pt nanomembranes. It is noteworthy that, compared to other Experimental Section, PSBEE is a low-cost, high yield, and easily implemented method we previously developed [24–26]. The simplicity of this method and the ultrathin thickness of the nanomembranes reduce costs in both production and materials, making our 2D Pt catalyst even more cost-effective than none-noble catalysts [27]. Furthermore, unlike other Pt catalysts, our Pt nanomembranes are made up of nanocrystals with a highly distorted atomic structure. Therefore, they exhibit outstanding HER performance with a small overpotential, good stability, and high turnover frequency (TOF). These advantageous features establish our Pt nanomembranes as a potentially viable alternative to the current state-of-the-art commercial Pt/C electrocatalyst [28]. Importantly, PSBEE can be used to produce other 2D metals at low costs, such as Au [29], Ag, Ti [30] and even high entropy alloys [31], hence enabling us to apply our current approach to other industrially essential catalytical reactions.

## Experimental Section

### Synthesis of Pt Nanomembrane

We fabricated Pt nanomembranes with thicknesses ranging from 5 to 28 nm using the polymer surface buckling-enabled exfoliation (PSBEE) method we previously developed [24–26]. First, we spin-coated a polyvinyl alcohol (PVA) hydrogel layer onto a glass plate and allowed it to dehydrate. Next, platinum was deposited onto the PVA layer using magnetron sputtering. The Pt-PVA-glass system was then immersed in deionized (DI) water. After several minutes, the freestanding nanomembranes spontaneously peeled off from the PVA substrate (Video S1).

### Structure and Electronic Properties Characterization

We employed atomic force microscopy (AFM, MFP-3D Origin, Oxford Instruments), X-ray photoelectron spectroscopy (XPS), and transmission electron microscopy (TEM) to characterize and measure the structural and chemical properties of our Pt nanomembranes. First, we transferred the freestanding Pt nanomembranes onto various substrates, such as Si wafers and TEM grids. We examined the thickness and surface topography of the as-prepared Pt nanomembranes on Si wafers using AFM, and those on grids using conventional field emission TEM (JEM-2100, JEOL) at an acceleration voltage of 200 kV and Cs-corrected thermal field emission TEM (ARM200F, JEM) at 300 kV. We employed Agilent 720ES (OES) to determine the content of Pt loading for different thicknesses and the Pt ion concentration after stability tests.

Data reduction, analysis, and EXAFS fitting were performed using the Athena and Artemis programs of the Demeter data analysis packages [32]. These programs utilize the FEFF6 program [33] to fit the EXAFS data. We conducted energy calibration for the sample using a standard Pt foil as a reference, which was measured simultaneously. A linear function was subtracted from the pre-edge region, and the edge jump was normalized using Athena software. We isolated the *χ*(*k*) data by subtracting a smooth, third-order polynomial approximating the absorption background of an isolated atom. The *k*3-weighted *χ*(*k*) data were Fourier-transformed after applying a Hanning window function (Δ*k* = 1.0). For EXAFS modeling, we obtained global amplitude EXAFS (CN, *R*, σ^2^, and *ΔE*_0_) through nonlinear fitting and least-squares refinement of the EXAFS equation to the Fourier-transformed data in R-space, using Artemis software. We fitted the EXAFS of the Pt foil and set the obtained amplitude reduction factor *S*_0_^2^ value (0.839) in the EXAFS analysis to determine the coordination numbers (CNs) in the Pt-C/O/Pt scattering path in the sample.

### Electrochemical Measurements and PEMWE Tests

In a typical test, the freestanding 2D Pt nanomembrane can be transferred to any substrate. After peeling off from PVA substrates, the Pt nanomembranes floated on the water surface. Following our previous established method [30], we immersed the carbon clothe beneath the water and selectively “scooped out” the desired number of Pt nanomembranes. By employing this approach, we successfully achieved controlled loading of Pt nanomembranes onto the commercial carbon cloth, which was subsequently dried at room temperature (Video S2). We performed all measurements at room temperature in a standard three-electrode system using an H_2_-saturated 0.5 M H_2_SO_4_ electrolyte. We used a carbon rod (diameter = 6 mm) as the counter electrode and a saturated calomel electrode (SCE) as the reference electrode. We calibrated the SCE to the reversible hydrogen electrode (RHE) under H_2_-saturated electrolyte with Pt foils serving as both the working electrode and counter electrode (Fig. S12). We conducted electrochemical impedance spectrum measurements at an overpotential of 10 mV versus. RHE with a 10 mV AC potential, ranging from 10^5^ to 0.01 Hz. We collected the time-dependent potential curve by maintaining the current density at 10 mA cm^−2^ for 24 h. The setup for PEMWE is described as follows: a serpentine flow field using *S*-type titanium was used as the bipolar plate to separate the two electrodes and collect current. The PEMWE electrolyzer was assembled in the following sequence: end plate, sealing gasket, titanium current collector, 19 nm Pt nanomembrane on carbon cloth, proton-exchange membrane, IrO_2_, titanium current collector, sealing gasket, and end plate. The anode consisted of IrO_2_ (99.9%, Macklin) with the mass loading of 0.43 mg cm^−2^. The total geometric area of both the cathode and anode was 4.0 cm^2^. A proton-exchange membrane (DuPont Nafion PFSA N117) was used to separate the cathode and anode compartments of the electrolyzer. For the test, 0.5 M H_2_SO_4_ electrolyte was supplied to both sides of the electrolyzer at a rate of 2.5 mL min^−1^, controlled by a peristaltic pump. We reported cell voltages measured in PEM–WE without IR compensation.

### TOF and Active-Site Density Calculations

The total number of hydrogen turnovers was calculated from the current density using formula [34]:

$$ {\text{TOF}} = \frac{{{\text{Total number of hydrogen turnover}}/{\text{ geometric area }}\left( {{\text{cm}}^{2} } \right)}}{{{\text{Number of active site}}/{\text{geometric area }}\left( {{\text{cm}}^{2} } \right)}} $$

$$ \begin{aligned} {\text{The number of hydrogen }} & = {\text{j}}\left( {\frac{{{\text{mA}}}}{{{\text{cm}}^{2} }}} \right)1\left( {{\text{Cs}}^{ - 1} \left( {10^{3} {\text{ mA}}} \right)^{ - 1} } \right)\left( {1{\text{ mole e}}^{ - } \left( {96,485.3{\text{ C}}} \right)^{ - 1} } \right) \\ \quad \times \frac{{1{\text{ mole H}}_{2} }}{{2{\text{ mole e}}^{ - } }}\frac{{6.022 \times 10^{23} {\text{ molecules H}}_{2} }}{{1{\text{ mole H}}_{2} }} \\ \quad = 3.12 \times 10^{15} {\text{ H}}_{2} {\text{ s}}^{ - 1} {\text{ cm}}^{ - 2} {\text{ per mA cm}}^{ - 2} \\ \end{aligned} $$

The Pt loading *L* was determined from the inductively coupled plasma optical emission spectroscopy (ICP–OES) measurement (Fig. S13f). Thus, the active site density on bulk Pt is:

$$ L \times \frac{{1\;{\text{mmol}}}}{{195.1\;{\text{mg}}}} \times 6.022 \times 10^{20} $$

Here, we calculate TOF at the potential of 100 mV with the current density for the 5-, 10-, and 19-nm-thick nanomembranes being 74.7, 83.8, and 89.7 mA cm^−2^, respectively.

$$ {\text{TOF}}_{{\text{5 nm}}} = { }\frac{{3.12 \times 10^{15} \times {\text{H}}_{2} {\text{ s cm}}^{2} \times 74.7{\text{ mA}}/{\text{cm}}^{2} }}{{1.6 \times 10^{15} }} = { 145}.{\text{7 s}}^{{ - {1}}} {\text{per Pt}}{-}{\text{site}} $$

$$ {\text{TOF}}_{{{1}0{\text{ nm}}}} = { }\frac{{3.12 \times 10^{15} \times {\text{H}}_{2} {\text{ s cm}}^{2} \times 83.8{\text{ mA / cm}}^{2} }}{{2.9 \times 10^{15} }} = { 9}0.{\text{2 s}}^{{ - {1}}} {\text{per Pt}}{-}{\text{site}} $$

$$ {\text{TOF}}_{{\text{19 nm}}} = { }\frac{{3.12 \times 10^{15} \times {\text{H}}_{2} {\text{ s cm}}^{2} \times 89.7{\text{ mA}}/{\text{cm}}^{2} }}{{5.8 \times 10^{15} }} = { 48}.{\text{3 s}}^{{ - {1}}} {\text{per Pt}}{-}{\text{site}} $$

### Cost Per Electrode Area

In evaluating the cost per electrode area, we have followed the commonly used practice of basing the estimation on the price of raw materials, which allows for meaningful comparisons [35, 36]. We assessed the Pt price for Pt-based catalysts, while for non-noble metal catalysts, we evaluated the price of all related metals. For Pt-containing catalysts, the price of raw materials such as H_2_PtCl_6_, MeCpPtMe_3_, and K_2_PtCl_4_ is denoted as *P*_R_ ($ g^−1^), the molecular weight of the raw material is *M*_R_, and the Pt loading is *m* (g cm^−2^). The price of the catalyst per area in the working electrode is calculated as *P* = *P*_R_ × *195 m/M*_R_. For non-noble metal catalysts, the mass of the catalyst per area in the electrode is *m* (g cm^−2^), the usage of raw material to fabricate the catalyst per unit mass is *m*_R_ (g g^−1^, i.e., dimensionless), and the raw material price is *P*_R_ ($ g^−1^). The price of the catalyst per area in the working electrode is calculated as *P* = *P*_R_ × *m* × *m*_R_. The detailed cost estimation process is summarized in Fig. S21. And the price of the raw materials is summarized in Tables S4 and S5.

### Mechanical Characterization

We characterized the mechanical properties of the prepared Pt nanomembranes using AFM-based indentation [37] (Fig. S18). We transferred the freestanding Pt nanomembranes onto patterned Si wafers and suspended them over the holes. We used a diamond-coated silicon tip (NC-LC, Adama) with a 30 nm tip radius to perform indentation measurements at a rate of 300 nm s^−1^. We tested Pt nanomembranes with thicknesses of 19 and 28 nm under ambient conditions, with each group containing 15 nanomembranes. We derived the Young’s modulus, yield strength, and ductility from these data sets.

According to Ref. [38], we extracted the Young’s modulus, yield strength, and ductility of Pt nanomembranes from finite element analysis (FEA) performed using the commercial software ANSYS (ANSYS Inc., USA). In the theoretical model, we modeled the suspended nanomembranes in actual experiments as an axisymmetric membrane with a radius of 1–2 µm. In the model, we used a rigid, frictionless sphere with a 30 nm radius to replace the AFM tip. We took the Poisson’s ratio of nanomembranes as 0.35, based on platinum. We traced back the Young’s modulus and yield strength using elastic and elastoplastic constitutive equations. Additionally, we obtained the ductility of Pt nanomembranes as the maximum von Mises strain developed right before strain softening.

### Theoretic Calculations

The first-principles total energy calculations were performed using the Vienna Ab initio Simulation Package (VASP) and the projector augmented wave (PAW) method [39]. A plane wave cutoff energy of 400 eV was employed. Convergence criteria for energy and force were set at $10^{ - 5}$ eV and 0.02 eV Å^−2^, respectively. A 4 × 4 supercell containing eight atomic layer was constructed to simulate models I and II of Pt (111), (110), and (100) incorporating a vacuum layer larger than 15.0 Å along the *z*-axis to prevent periodic interaction (inset of Fig. 4d). Tensile strain ranging from 0 to 7% was applied along the *x*-axis, which increased the elastic strain in the unit cell correspondingly. In this unit slab cell, vacancy concentrations were incremented by 6.25% (up to 18.75%), with vacancy concentration defined as the total number of vacancies divided by the total number of atoms in the pristine basal plan.

The Gibbs free energy of H adsorption ($\Delta G_{{{\text{H}}^{*} }}$), a widely used descriptor for correlating theoretical predictions with experimental measurements of catalytic activity for various systems [40, 41], was calculated in a manner consistent with previous studies [42]. The following equation was used:

$$ \Delta G_{{{\text{H}}^{*} }} = {\Delta }E_{{{\text{H}}^{*} }} + \Delta E_{{{\text{ZPE}}}} - T\Delta S $$

where $\Delta E_{{{\text{H}}^{*} }}$ denotes the hydrogen adsorption energy, $\Delta E_{{{\text{ZPE}}}}$ represents the zero-point energy correction term, *T* is the temperature (298.15 K), and *ΔS* represents the entropy difference. Optimal catalytic activity is indicated by a $\Delta G_{{{\text{H}}^{*} }}$ value close to zero, with very negative or positive values signifying overly strong or weak adsorption, respectively.

The large-scale atomic/molecular massively parallel simulator (LAMMPS) package [33] was utilized to perform MD simulations. In these simulations, an empirical force field was described using a modified embedded-atom method (MEAM) potential for the Pt-C system developed by Jeong et al. [34]. To observe the lattice distortion effect, approximately 20% of C atoms were introduced as substantial defects in Pt nanocrystals, which had a nanograin size of approximately 10 nm. The atomic configurations were visualized using the OVITO software [35].

## Results and Discussion

### Structural Characterization

**Fig. 1:**
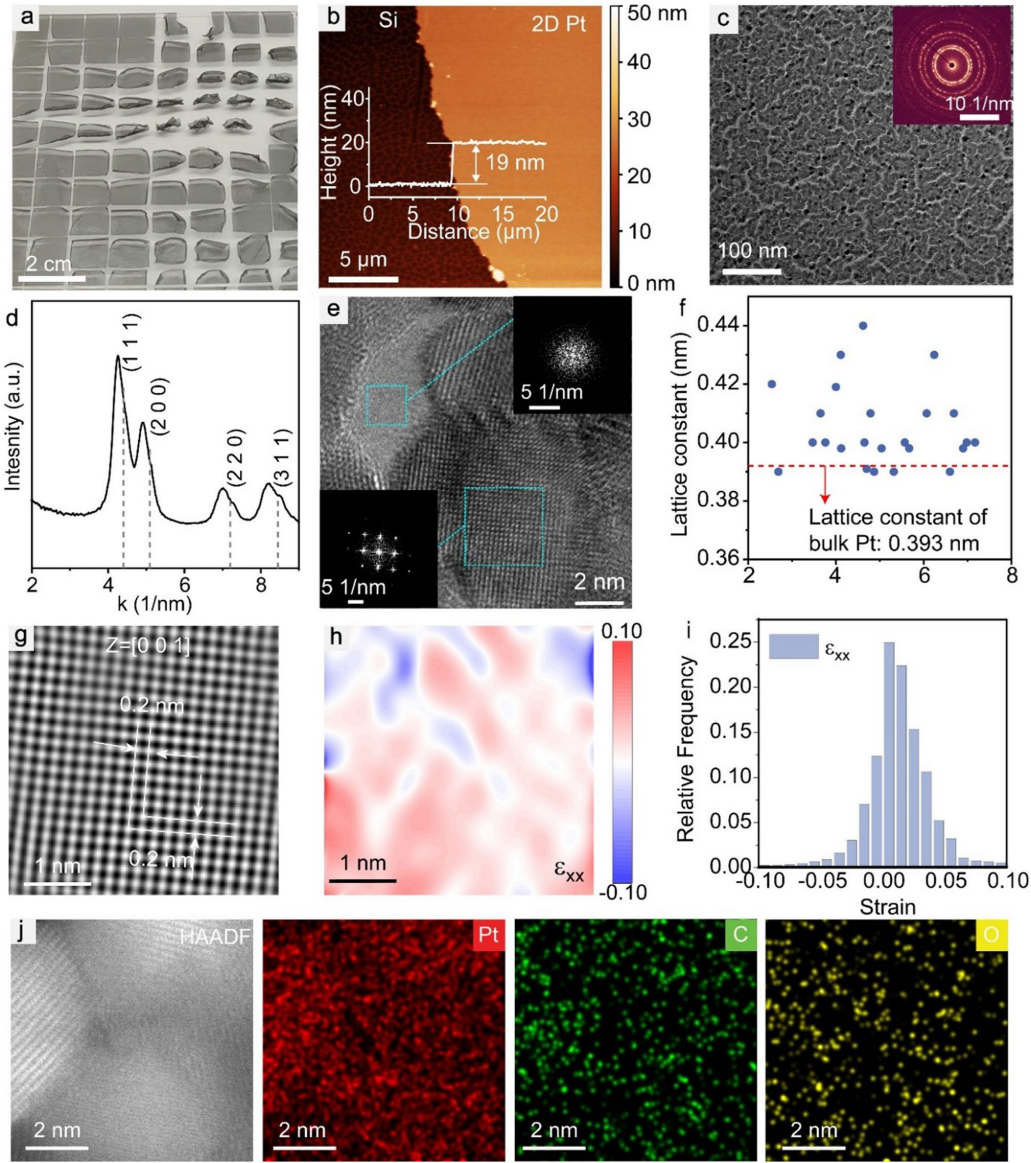
Structural characterization of our freestanding Pt nanomembrane. **a** Photograph of the 19-nm-thick freestanding Pt nanomembranes on water surface. **b** Height profile image of a transferred 19-nm-thick Pt nanomembrane on silicon; the inset shows the AFM image across the edge of the Pt nanomembrane. **c** Low-magnification TEM image of the 19-nm-thick freestanding Pt nanomembrane, inset shows the corresponding SADP. **d** Radially integrated intensity of the diffraction patterns of the freestanding nanomembrane, in comparison with those of the single-phase FCC bulk Pt as indicated by the dash lines. **e** The high revolution TEM image of the 19-nm-thick Pt nanomembrane. Insets are fast Fourier transform (FFT) patterns of the amorphous (upper right) and crystalline (lower left) regions. **f** Lattice constant with the size of Pt nanocrystal (NC). **g** Inverse fast Fourier transformation (IFFT) analysis of the high-resolution TEM image of a typical Pt NC with a FCC structure in (**e**). **h** Contour map of the normal stain ɛ_xx_ in (**g**). **i** Distribution of the strain ɛ_xx_ component. **j** High-angle annular dark-field scanning transmission electron microscopy (HAADF-STEM) image of a typical Pt NC with a FCC structure and corresponding elements maps of Pt, C, and O

In line with PSBEE [24–26], we successfully fabricated a series of large area, freestanding 2D Pt nanomembranes, as displayed in Fig. 1a (see Video S1 and also Experimental Section for details). The thicknesses of these 2D Pt can be easily varied from 5 to 28 nm, while the in-plane size remains at approximately 1 cm (Figs. 1b and S1a–c). We performed XRD characterization on our Pt nanomembrane. As anticipated, the results demonstrate that the diffraction peaks align with those of bulk FCC Pt and exhibit considerable broadening (Fig. S2). However, we would like to highlight that, as discussed in our recent review article [43], the interpretation of peak broadening in metal XRD spectra encompasses various factors, such as lattice strain, dislocations, and grain boundaries. Therefore, direct observations using TEM still remain a reliable method to characterize lattice distortion in metals. A low-resolution TEM image of our Pt nanomembrane, shown in Fig. 1c, shows a heterogeneous nanostructure aligned to FCC atomic packing as revealed in the corresponding selected-area diffraction pattern (SADP) (inset of Fig. 1c). Using the 2D SADP and integrating it with respect to the diffraction angle [44], we successfully computed a series of 1D diffraction peaks, which align with a typical diffraction pattern of FCC, except for a noticeable peak shift to a lower wave number (Fig. 1d). This shift indicates that compared to bulk Pt, there exists an overall tensile strain in the FCC lattices within our Pt nanomembranes.

Figure 1e displays high-resolution TEM (HRTEM) of our Pt nanomembranes, revealing the presence of amorphous regions between clusters of FCC nanocrystals (NC). STEM-EDS analysis (Fig. 1j) showed that the FCC NCs primarily contain Pt with C and O elements. Through extensive quantitative measurements, we observed that the size of the Pt NCs ranged from 3 to 7 nm, with significant lattice expansion compared to bulk Pt (Fig. 1f). By utilizing geometric phase analysis (GPA) [45], we were able to map out the lattice strains in the Pt NCs that are free of crystalline defects, such as dislocations (Figs. 1g-i and S3a–c). Figures 1i and S3b, c exhibit the lattice strain component distribution obtained using GPA. Evidently, the lattice strain field within our Pt NCs is heterogeneous, consisting of both shear and normal components, which is similar to the heterogeneous lattice strain fields found in high entropy alloys [45]. Notably, the averaged normal strains are positive or tensile, consistent with the average lattice strain obtained by measuring lattice constants (Fig. 1d, f). Our previous studies [29, 31] suggest that creating metallic nanomembranes via PSBEE (e.g., gold [29] and high entropy alloy [31]) can be regarded as assemblies of metallic NCs during metal deposition and exfoliation. Similarly, our Pt nanomembranes display a heterogeneous nanostructure that features NC assembly and percolation. The TEM analysis of the 5 nm Pt nanomembrane reveals the presence of amorphous carbon and dispersed Pt nanoparticles, as observed in Fig. S4. Due to the gradual transition from the amorphous carbon to the crystalline Pt, we expect a significant lattice distortion, particularly in the near-interface region. However, quantitative characterizing the strain is challenging due to the signal mixing from the amorphous and crystalline regions. In contrast, when the nanomembrane thickness increases to 10 nm and the nanoparticles percolate to form a network structure (Fig. S5), we can employ our previously established approach to characterize the lattice strains in the 10 nm Pt nanomembrane. These strains are similar to those obtained for the 19 and 28 nm Pt nanomembranes. Additionally, we have observed a notable presence of vacancies in the Pt nanomembrane. Furthermore, we conducted conductive AFM (C-AFM) scanning of our Pt nanomembranes. From Fig. S1d, we could infer that our Pt nanomembrane demonstrates a heterogeneous distribution of local electric conductivity, with highly conductive nano-domains surrounded by less conductive ones. Such conductivity distribution manifests the heterogeneous nanostructure of our Pt nanomembranes.

### Electronic Properties of Pt Nanomembrane

**Fig. 2:**
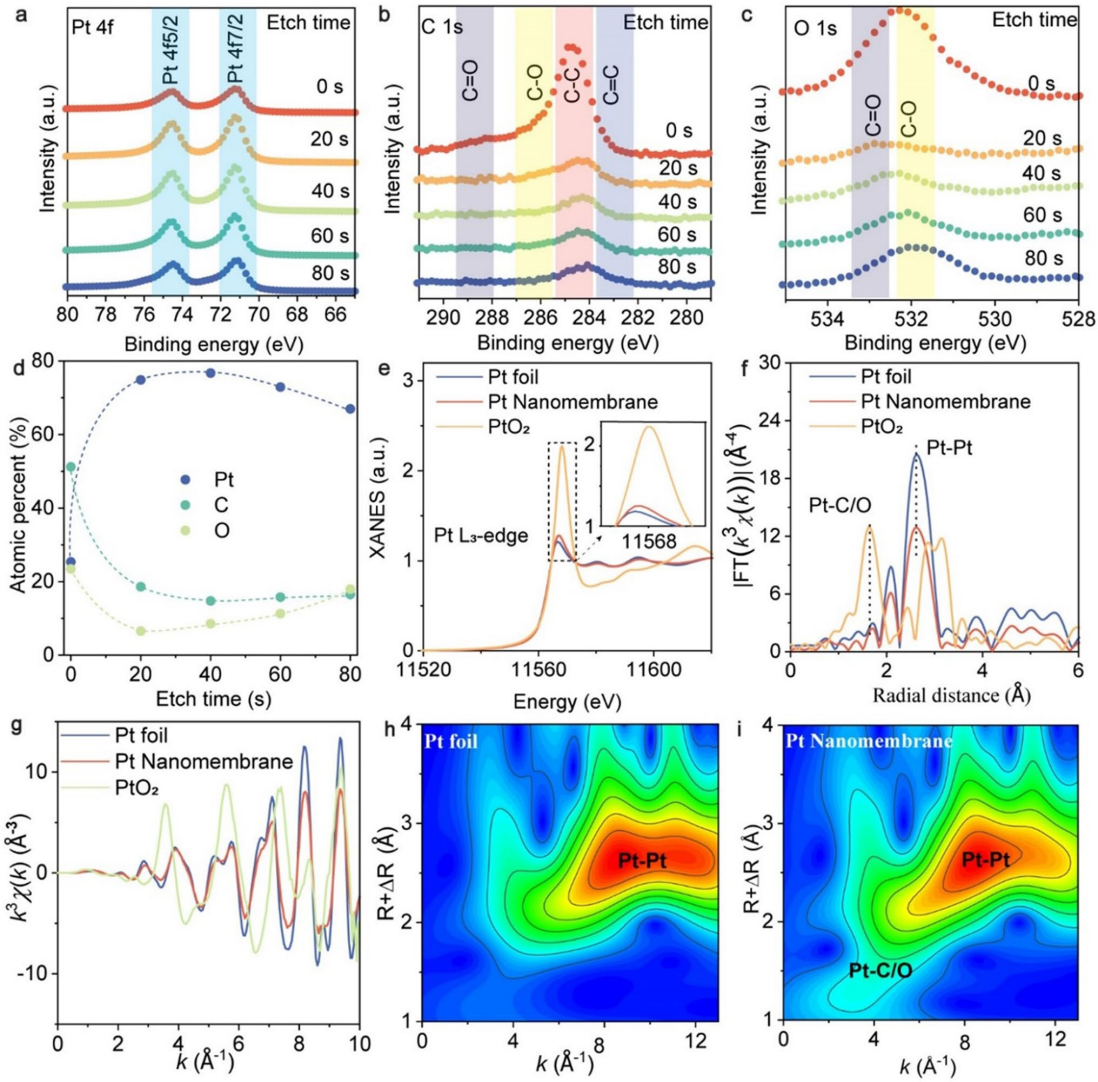
Chemical bonding and atomic packing analyses of our 19 nm Pt nanomembranes. **a**–**c** X-ray photoelectron spectroscopy (XPS) depth profile analysis of Pt nanomembrane. Narrow-scan XPS spectra for **a** Pt 4*f*, **b** C 1*s*, and **c** O 1*s* as a function of etching time. **d** Relative atomic concentration of Pt, C, and O with etching time, as obtained from the quantitative analysis of the XPS. **e** X-ray absorption near edge structure (XANES) at Pt L_3_ edge. **f** FT-EXAFS region for the local structure of Pt. **g** EXAFS *χ*(*k*) signals in *k*-space. **h** and **i** show the wavelet transform for the *k*_3_-weighted EXAFS Pt *K*-edge signal of Pt foil and Pt nanomembrane, respectively

**Fig. 3:**
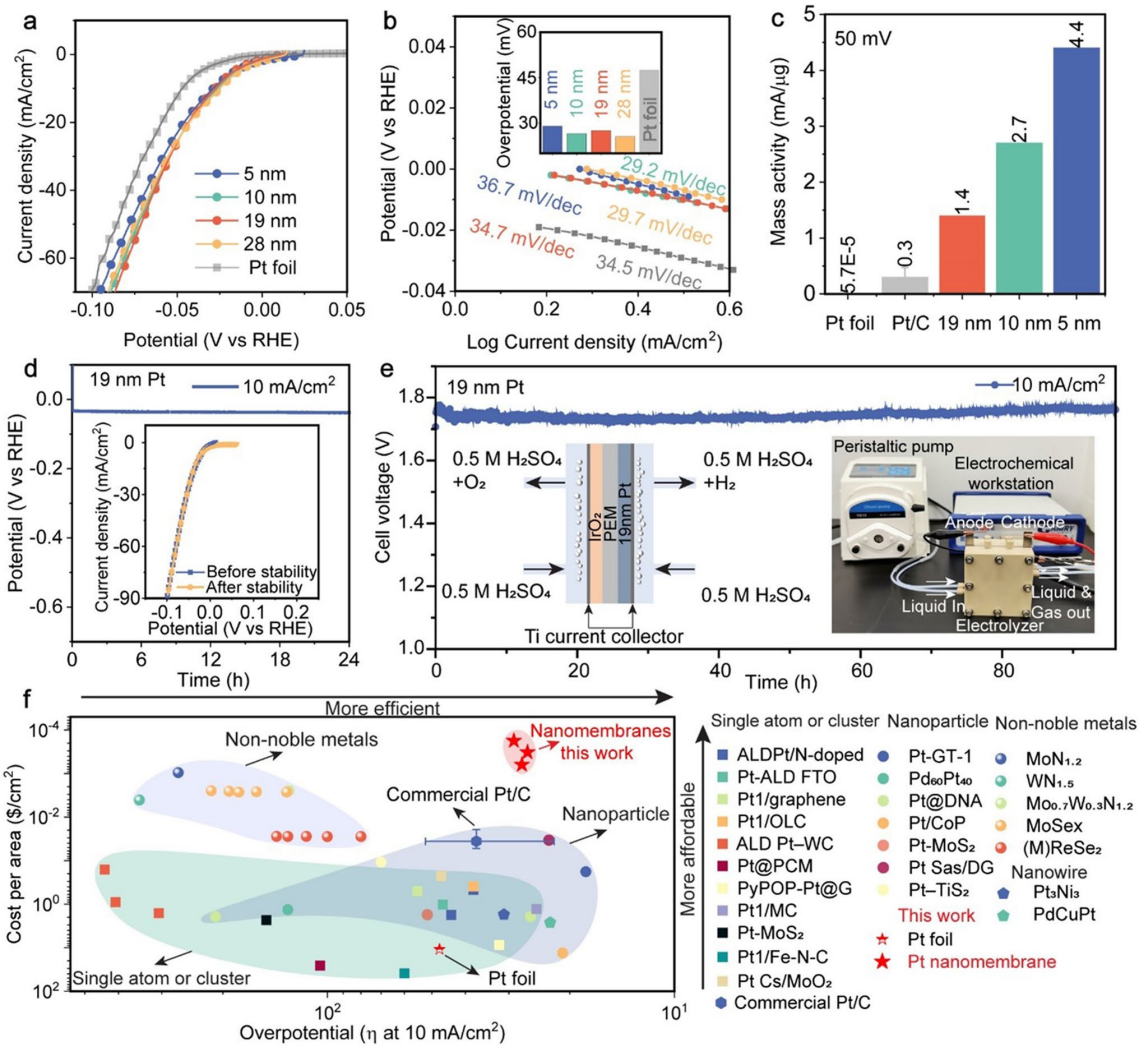
Acidic HER performance of our Pt nanomembranes. **a** Linear sweep voltammetry (LSV) curves, **b** Tafel slopes of 5-nm, 10-nm, 19-nm, 28-nm-thick Pt nanomembrane and bulk Pt foil. The inset shows overpotential at 10 mA cm^−2^. **c** Comparison of mass activities measured at 50 mV for our Pt nanomembranes with those of Pt foil and commercial Pt/C. **d** Stability tests of 19-nm-thick Pt nanomembrane at the current density of 10 mA cm^−2^. **e** Proton exchange membrane water electrolyzer (PEM–WE) device performance using our 19 nm Pt nanomembrane as the cathodic HER catalyst and Commercial IrO_2_ as the anodic OER catalyst at room temperature, insets are the schematic (left) and photograph (right) of the PEM–WE. **f** Comparison of our Pt nanomembranes and other HER electrocatalysts in terms of overpotential at 10 mA cm^−2^ and cost per area

To further characterize the chemistry of our Pt nanomembranes, XPS was conducted (Fig. 2a-d). The observed XPS spectra for Pt, C, and O after various etching times showed two prominent peaks at 71.1 and 74.5 eV that correspond to Pt^0^ in its metallic state (Fig. 2a). The XPS spectrum of C 1*s* (Fig. 2b) showed four distinct peaks at approximately 282.9, 284.8, 286.2, and 288.5 eV, which were attributed to C=C, C–C, C–O, and C=O, respectively. As previously reported in our studies [29, 31, 46], these chemical bonds were expected to be residuals from the decomposed PVA during the exfoliation process of 2D metals. The XPS spectrum of O 1*s* (Fig. 2c) exhibited two main peaks at approximately 532.2 and 533 eV, corresponding to C–O and C=O, respectively, which provides further evidence of decomposed PVA. We have performed fitting of the XPS spectra, and the results are presented in Figs. S7 and S8. Based on our fitting analysis (Figs. S7a, c and S8a, c), we did not observe any indications of Pt oxides. However, we did observe that the distributions of binding energy for Pt^0^ exhibit a skewness toward high energy values, leading to the presence of fat tails as depicted in Fig. S7a, d. This observation could potentially be attributed to lattice distortion within the Pt nanocrystals. Through data fitting (Fig. S7), we show the relative concentrations of Pt, C, and O as a function of etching time (Fig. 2d), which demonstrates the chemical gradient in our Pt nanomembrane. The variation in composition with etching time is primarily due to the formation of a gradient nanostructure resulting from the reactions of metals and PVA. This phenomenon of a gradient nanostructure has also been observed in gold nanomembranes fabricated using PSBEE [29]. Moreover, X-ray absorption spectroscopy (XAS) was performed on the Pt nanomembrane as well as two reference materials (e.g., Pt foil and PtO_2_) (Fig. 2e). Owing to the presence of metallic Pt, the Pt *L*_3_ edge threshold energy and maximum energy for the X-ray absorption by our Pt nanomembrane look quite similar to those of Pt foil. However, upon closer inspection, it is evident that the white line intensity of our Pt nanomembrane is slightly higher than that of Pt foil (Pt^0^), but significantly smaller than that of PtO_2_ (Pt^IV^). This behavior implies that, despite the dominant metallic Pt bonding, there is a tendency to form a cationic environment around Pt [15, 23]. The Fourier transforms of the extended X-ray absorption fine structure (EXAFS) region yielded two prominent peaks at approximately 2.7 and 1.7 Å, corresponding to Pt–Pt and Pt–C/O coordination, respectively (Fig. 2f) [14]. The EXAFS analysis (Fig. S9a–d and Table S1) revealed an average Pt coordination number of approximately 9.8 for our Pt nanomembrane, which is smaller than the conventional Pt coordination number (= 12), indicative of a defected atomic structure in the Pt nanomembrane. Figure 2g shows the curves of EXAFS *χ*(*k*) signals versus in *k*-space obtained for our Pt nanomembrane, PtO_2_ and Pt foil, from which we can see that the Pt nanomembrane is similar to Pt foil. Aside from Pt–Pt bonds, a few Pt–C/O bonds were observed in the nanomembrane (Fig. 2h, i). Notably, similar results were observed for the 28-nm-thick Pt nanomembrane (Figs. S8 and S10). Due to the limitation of the EXAFS technique, which does not allow for a direct distinction between Pt–C and Pt–O, it is challenging to measure their coordination numbers. To obtain an indirect estimation, we referred to the XPS data (Fig. 2d), which revealed a relatively higher atomic percentage of C compared to O. Based on this observation, it is plausible that the coordination number of Pt–C is larger than that of Pt–O. Following this line of reasoning, we anticipated that, with the thickness decreases, the coordination number of Pt–C/O would increase, as supported by the EXAFS data of 19 and 28 nm Pt nanomembranes (Table S1). However, it is important to note that there is no clear trend, indicating that the Pt–C/O ratio significantly affects the HER performance, as depicted in Fig. 3.

### Electrochemical Characterization in Acidic Electrolyte

For the evaluation of their electrocatalytic properties, we transferred our Pt nanomembranes onto commercial carbon clothes (Video S2). As shown in Fig. S11, due to the Van der Waals force, our nanomembranes were wrapped around individual carbon fibers in the carbon cloth after the nanomembrane transfer. A three-electrode system in 0.5 M H_2_SO_4_ was then set up for HER. Figure 3a shows that, as an electrocatalyst, our Pt nanomembranes exhibit negligible onset potentials, outperforming bulk Pt foil. From the polarization curves, we extracted *η*10 (i.e., the overpotential at 10 mA cm^−2^) and the Tafel slopes for our Pt nanomembranes with four different thicknesses (Fig. 3b). Notably, *η*10 of our Pt nanomembrane catalysts ranges from 26 to 29 mV, which is much smaller than that (= 46 mV) of Pt foil catalysts. The Tafel slopes of our Pt nanomembrane catalyst ranges from 30 to 37 mV dec^−1^, suggesting a mechanism combining the Volmer–Tafel and Volmer–Heyrovsky reaction [47]. Likewise, the electrochemical impedance spectroscopy (EIS) and cyclic voltammetry (CV) results also show that the HER performance of our nanomembranes is better than that of Pt foil (Figs. S13b and S14). We also compared our Pt nanomembranes with other electrocatalysts reported so far with regard to *η*10 and the Tafel slope. As shown in Fig. S13g**,** the performance of our Pt nanomembranes is among the best. Moreover, since mass activity and turnover frequency (TOF) are another important metrics to assess the potential scalability of metal-based electrocatalysts [48], we measured the catalyst loading of our Pt nanomembranes by inductively coupled plasma optical emission spectroscopy (ICP-OES) (Fig. S13f) and calculated their mass activities at the overpotential of 50 mV. As shown in Fig. 3c, the mass activities of our Pt nanomembranes increase from 1.4 for 19-nm-thick nanomembranes to 4.4 for 5-nm-thick nanomembranes, which is about five orders of magnitude higher than that of bulk Pt foil (5.7 × 10^–5^) and one order of magnitude higher than that of commercial Pt/C (0.3). We further quantify the catalytic efficiency of each Pt site with TOF, as shown in Table S3, the TOF values at the overpotential of 100 mV range from 48.3 to 145.7 S^−1^ for our Pt nanomembranes, significantly higher than those of most HER catalysts.

Following Refs. [10, 15, 49], we evaluated the electrocatalytic stability of our Pt nanomembranes at the current density of 10 mA cm^−2^ (Figs. 3d and S13c–e) and did not see any significant potential amplification for 24 h. We performed a comparison of the LSV curves for the nanomembranes with varying thicknesses before and after the stability tests. Remarkably, these curves exhibited a high degree of overlap, indicating excellent stability (inset of Figs. 3d and S13c–e). Besides, we examined the morphology of our Pt nanomembrane and Pt concentration in the electrolyte after the stability test. As shown in Fig. S15, our Pt nanomembrane still adhered to the carbon fibers after the long-term test, and the Pt ion concentration in the solution was negligible (2.2 µg L^−1^). We conducted extensive XPS analysis following the stability tests lasting 24 h at the current density of 10 mA cm^−2^. Remarkably, we consistently observed two prominent peaks at 71.1 and 74.5 eV, which correspond to the metallic state (Pt^0^) of Pt, regardless of the etching time. Furthermore, the O 1*s* analysis revealed no indications of metal oxides or any peaks within the 529–530 eV range, providing further evidence of the excellent electrochemical stability of the Pt nanomembranes. However, it is worth noting that we observed significant skewness in the binding energy distributions for Pt^0^ toward high energy values, suggesting possible further distortion of the Pt nanocrystals after the stability tests. Additionally, we examined the carbon clothes using SEM after the stability tests. The SEM results revealed that most Pt nanomembrane remained adhered to the carbon fibers, with some microscale cracks. Note that with the decrease of thickness, Pt nanomembrane (5 nm) tends to fracture in a brittle mode, which makes it difficult for transfer without extensive premature failure. As a demonstration for future industrial applications, we chose 19 nm Pt nanomembranes as the cathodic HER catalyst and Commercial IrO_2_ as the anodic OER catalyst in a PEM–WE device because of easy transfer of 19 nm Pt nanomembrane. At the current density of 10 mA cm^−2^, the voltage remained to be a constant of ~ 1.72 V without IR compensation, and the overpotential amplification was negligible even after 100 h of testing. To evaluate their commercial viability, we calculated the cost per area of our Pt nanomembranes, and other catalysts reported in the literature (see Experimental Section, Tables S4 and S5 for details). As shown in Fig. 3f, it is evident that our Pt nanomembranes are several orders of magnitude cheaper than the Pt catalysts of other forms, including Pt foil, Pt nanoparticles, single Pt atom or cluster and commercial Pt/C. Owing to their ultrathin thickness, our Pt nanomembranes are even more cost-effective than non-noble metallic HER catalysts. Meanwhile, our Pt nanomembranes also show excellent catalytic efficiency, and its *η*10 is only slightly higher than that of a few single Pt atom or cluster catalysts but smaller than other HER catalysts, including the commercial Pt/C and non-noble metal catalysts.

### Atomistic Mechanisms

In previous studies, it has been demonstrated that elastic strain can enhance electrochemical catalytical reactions by modifying the binding energy of intermediates through changes in the electronic state at the Fermi level [41, 50]. However, these investigations have primarily focused on the effects of uniform strains applied to ideal crystals [51–53]. In contrast, our Pt nanomembrane is composed of highly distorted nanocrystals with heterogeneous elastic strains. Using atomic force microscopy (AFM) indentation (see Experimental Section), we found that the elastic modulus of the Pt nanomembrane was as low as 16 ± 2 GPa (Fig. S18), which is about 9% of the elastic modulus (= 179 GPa) of Pt nanocrystals with a size of 5 nm [54]. A similar phenomenon of lattice distortion-induced modulus reduction was also observed in high entropy alloys [55]. To understand the origin of such heterogeneous strains, we performed large-scale molecular dynamics (MD) simulations on Pt nanocrystals with a significant amount of substitutional C atoms (see Experimental Section). As demonstrated in Fig. S19, we observed a heterogeneous distribution of both shear and normal strains on the surface of the nanocrystals, induced by excessive crystalline defects (i.e., vacancies) on the surface and within nanocrystalline boundaries (Figs. S3, S5 and Table S1), resulting in surface strain distributions similar to our experimental results (Fig. 1h, i).

**Fig. 4:**
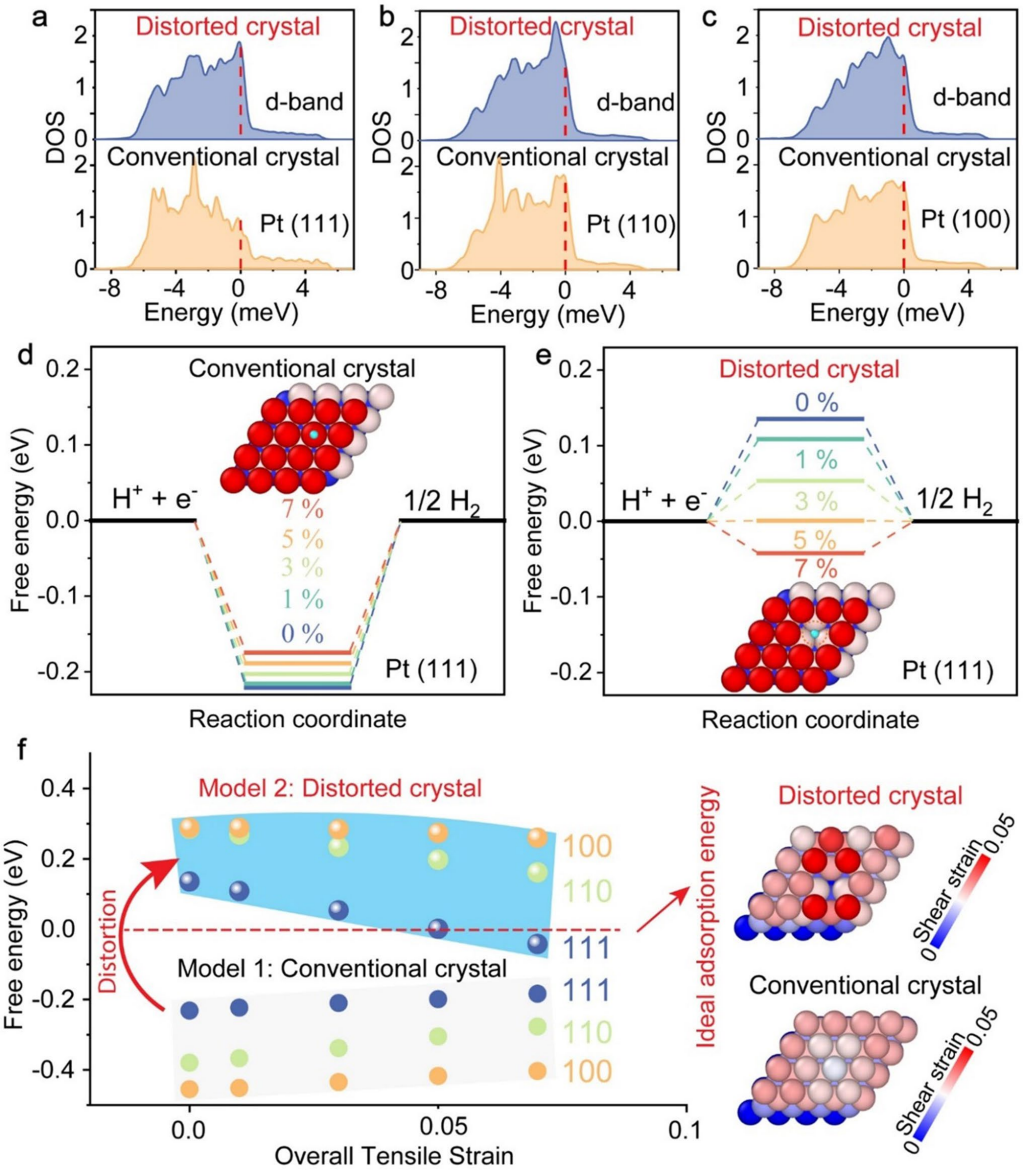
The atomic and electronic origin of excellent HER activity on our Pt nanomembrane made up of severely distorted nanocrystals. **a**–**c** Projected density of states (DOS) on pristine Pt and distorted Pt surface at crystal orientation (111), (110), and (100), respectively, the dot line indicates Fermi level. **d** Adsorption free energy versus the reaction coordinate of HER for a conventional crystal with the uniform tensile strain ranging from 0 to 7%. **e** Adsorption free energy versus the reaction coordinate of HER for a distorted crystal with the heterogeneous tensile strain of 0–7%. **f** Comparison of free energy vs strain between a conventional and a distorted crystal with the orientation of (111), (110), and (100), insets are the strain mappings of a distorted crystal and conventional crystal at the orientation (111)

Extensive density functional theory (DFT) simulations were performed to investigate heterogeneous strains on the surface adsorption behavior of distorted or defected nanocrystals. We systematically study the effect of heterogeneous strain. The reference model “ideal Pt nanocrystal” was subjected to a uniform strain from 0 to 7% in the (111), (100), and (110) orientations. Another atomic model was built to mimic lattice distortion with heterogeneous strains by inserting vacancies on the surface of Pt nanocrystals (see Experimental Section). The defected Pt nanocrystals were also subjected to a tensile strain from 0 to 7% along the (111), (100), and (110) orientations. As shown in Fig. 4a–c, the simulations revealed that heterogeneous strains in distorted nanocrystals resulted in an upshift of d-band centers, promoting H adsorption regardless of the crystal orientation [41]. The change in the Gibbs free energy *ΔG*_H_* for hydrogen adsorption on different surfaces was then calculated. As seen in Fig. 4d–f, *ΔG*_H*_ remained negative for all conventional Pt nanocrystals and increased with uniform strain. In sharp contrast, *ΔG*_H*_ turned positive when the Pt nanocrystals became severely distorted. As the distorted Pt nanocrystals were strained, *ΔG*_H*_ reduced progressively and even reached the zero energy at a strain of 5% on the distorted (111) surface, which was ideal for hydrogen production [42], but was never reached by simply straining conventional Pt within the Lindeman strain limit (~ 10%), above which a crystal becomes unstable [56]. These important findings indicate that lattice distortion, which manifests as heterogeneous elastic strains (the inset of Fig. 4f), can help regulate the hydrogen adsorption energy on the Pt surface over a much wider range than conventional elastic strain engineering, leading to a larger tunability of the HER performance. These computational results rationalize our experimental findings that distorted Pt nanocrystals are more efficient than conventional Pt nanocrystals in catalyzing water splitting. In our investigation, we also considered heteroatom doping, such as carbon doping or oxygen doping. Initially, an atomic model representing an ideal or no-distorted Pt nanocrystal was constructed as a reference. However, the results revealed that doping of carbon or oxygen did not have a beneficial effect on the HER performance (Fig. S20).

In recent years, 2D catalytic nanomaterials, including single atomic layers and nanomembranes, have emerged as a promising field of research, attracting significant interest due to their unique catalytic performance that can tailored by strain engineering [41], defect engineering [57, 58], and interface engineering [59]. However, the preparation of 2D catalysts often involves complex processes with low production rates and high costs compared to conventional bulk catalysts [60]. In contrast, we have demonstrated that the PSBEE method is a simple, scalable process capable of producing large quantities of metal-based nanomembranes (i.e., Au [29], high entropy alloy [31], and Pt) at a low cost, making it promising for the synthesis of next-generation low-dimensional electrocatalysts. Most importantly, unlike other 2D and conventional electrocatalysts, our Pt nanomembranes synthesized via PSBEE possess a unique nanostructure composed of severely distorted nanocrystals. The abundance of atomic-scale defects (i.e., vacancies) caused by lattice distortion and the resulting heterogeneous strains work synergically to enhance the HER catalytic performance of our Pt nanomembranes, surpassing commercial and many other conventional Pt electrocatalysts (Fig. 3f). Notably, this defect-strain synergy was also recently observed in MoS_2_ [53]_._ In addition to efficiency, our Pt nanomembranes exhibit excellent electrochemical stability in hydrogen production, showing negligible potential amplification at 10 mA/cm^2^ for approximately 100 h. However, it has been reported that low-dimensional electrocatalysts (e.g., RuO_2_, IrO_2_) may experience mechanical failure at high electric current densities [61], potentially due to the break-up of a large quantity of hydrogen bubbles near the electrode surface. According to Ref. [62], bubble break-up near a solid–liquid interface generates local shock waves and high impact forces, resulting in surface erosion in bulk catalysts [61] and most likely, premature failure of 2D catalysts. This common challenge calls for further research into the development of low-dimensional catalytical materials with high impact resistance, although this is beyond the scope of the current work.

## Conclusions

In conclusion, we developed ultra-efficient and affordable Pt nanomembrane HER electrocatalysts with an excellent electrochemical stability through PSBEE. With lattice distortion-induced heterogeneous strain in the Pt nanomembrane, HER performance enhancement was achieved. Besides, owing to the ultrathin nature of Pt nanomembrane, it is highly cost-effective and even comparable to non-noble metal catalysts. As PSBEE can be used to fabricate various metallic and ceramic nanomembranes regardless of their chemical compositions [26], we anticipate that our current approach may be applicable for developing low-dimensional electrocatalysts for many other crucial electrochemical reactions.

## Supplementary Information

Below is the link to the electronic supplementary material.

Supplementary file1 (PDF 4110 KB)

Supplementary file2 (MP4 7021 KB)

Supplementary file3 (MP4 6195 KB)
