# Supplementary material for: Ultra-Efficient and Cost-Effective Platinum Nanomembrane Electrocatalyst for Sustainable Hydrogen Production
Source: Nanomicro Lett. 2024 Feb 5;16:108. doi: 10.1007/s40820-024-01324-5 (PMC10844191; doi:10.1007/s40820-024-01324-5)
Supplement: Supplementary file 1 — Supplementary file1 (PDF 4110 KB) [file 40820_2024_1324_MOESM1_ESM.pdf]

Supporting Information for

Ultra-Efficient and Cost-Effective Platinum Nanomembrane Electrocatalyst for Sustainable Hydrogen Production

Xiang Gao[1, †], Shicheng Dai[1,2,3, †], Yun Teng[1, †], Qing Wang[4], Zhibo Zhang[1], Ziyin Yang[1], Minhyuk Park[1], Hang Wang[1], Zhe Jia[5], Yunjiang Wang[2,3], Yong Yang[1,6,*]

[1] Department of Mechanical Engineering, College of Engineering, City University of Hong Kong, Tat Chee Avenue, Kowloon Tong, Kowloon, Hong Kong, P. R. China

[2] State Key Laboratory of Nonlinear Mechanics, Institute of Mechanics, Chinese Academy of Sciences, Beijing, P. R. China

[3] School of Engineering Science, University of Chinese Academy of Sciences, Beijing, P. R. China

[4] Laboratory for Microstructures, Institute of Materials, Shanghai University, Shanghai, P. R. China

[5] School of Materials Science and Engineering, Jiangsu Key Laboratory for Advanced Metallic Materials, Southeast University, Nanjing, P. R. China

[6] Department of Materials Science and Engineering, College of Engineering, City University of Hong Kong, Tat Chee Avenue, Kowloon Tong, Kowloon, Hong Kong, P. R. China

† Xiang Gao, Shicheng Dai and Yun Teng contributed equally to this work.

*Corresponding author. E-mail: yonyang@cityu.edu.hk (Yong Yang)

Supplementary Figures and Tables

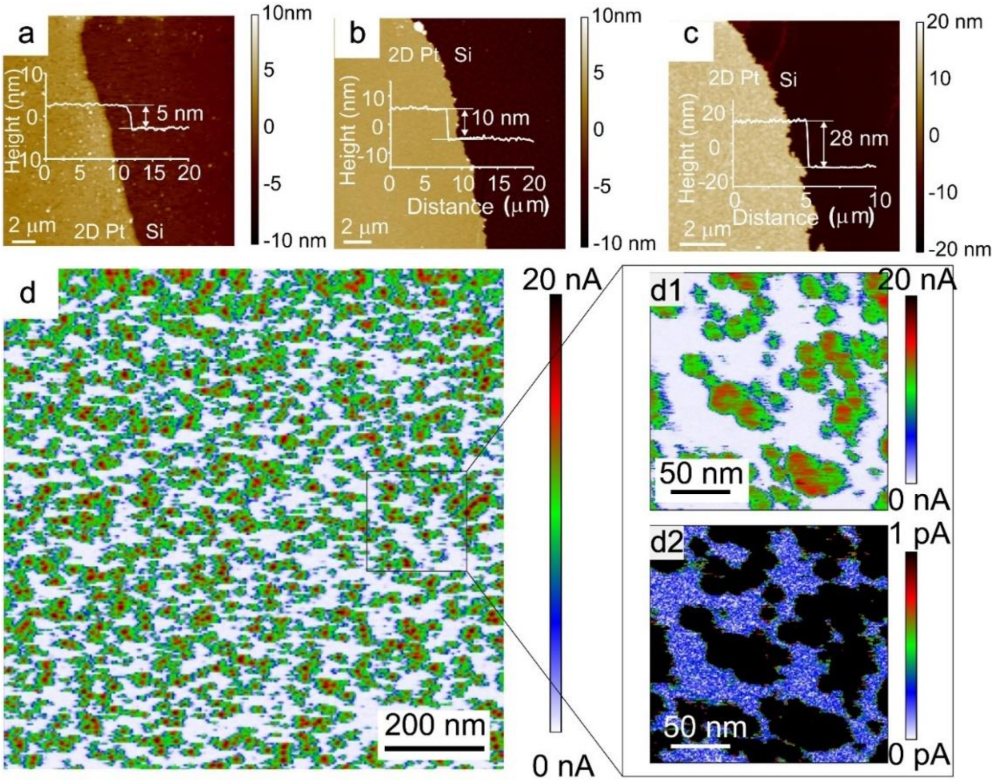

Fig. S1 Atomic Force Microscope (AFM) thickness measurement of (**a**) 5 nm, (**b**) 10 nm, (**c**) 28 nm. (**d**) Conductive atomic force microscopy (C-AFM) mapping image of 19 nm nanomembrane, inset figures show the enlarged views with different scale bars

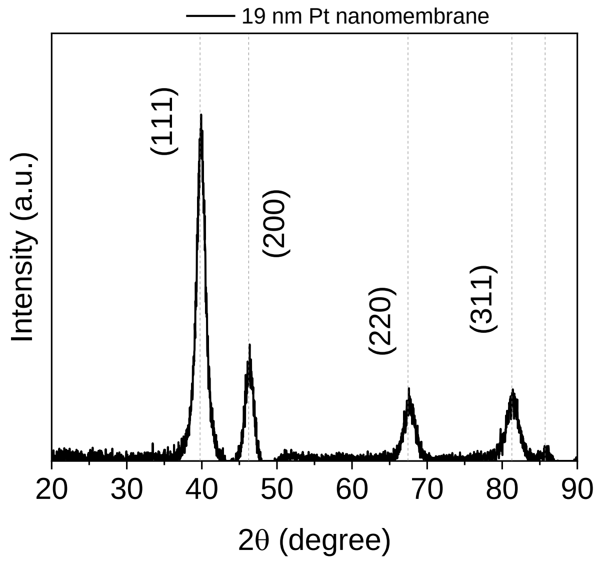

Fig. S2 X-ray diffraction pattern of 19 nm Pt nanomembrane

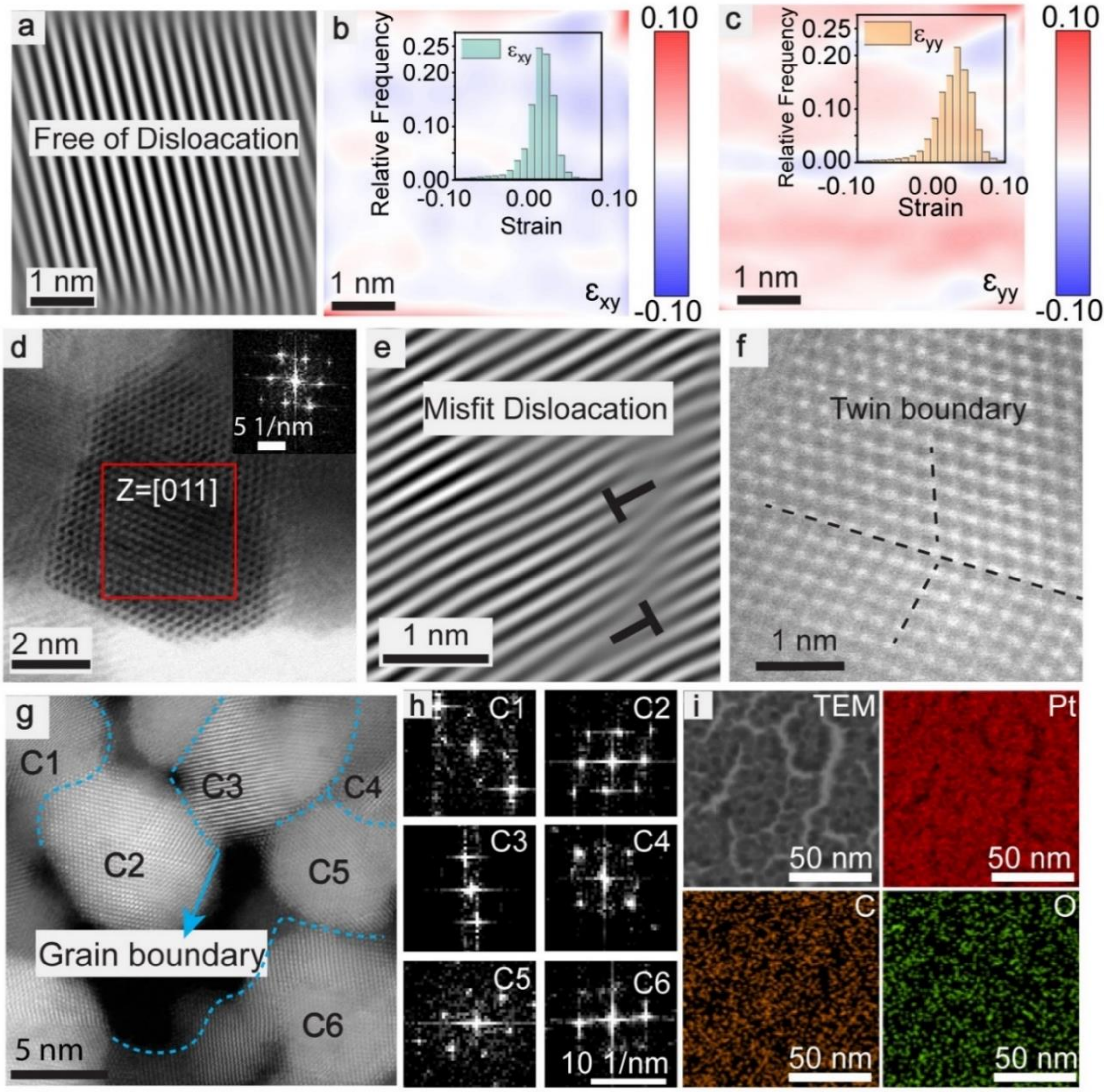

Fig. S3 Transmission electron microscope (TEM) analysis of 19 nm Pt nanomembranes. (**a**) The filtered IFFT image of Fig. 1g, showing a defect-free region. (**b**) Contour map of the normal strain ε_{xy} in Fig. 1**g**, inset is the distribution of the strain ε_{xy} component. (**c**) Contour map of the

normal strain ε_{yy} in Fig. 1**g**, inset is the distribution of the strain ε_{yy} component. (**d**) High-angle annular dark-field scanning transmission electron microscopy (HAADF-STEM) images from the [011] direction of a typical Pt nanocrystal (NC) with a FCC structure. (**e**) The filtered IFFT image of the region in Fig. S2d. (**f**) twin boundary. (**g**) Representative high-resolution STEM images of the Pt nanomembranes, the dashed line points out the grain boundaries. (**h**) The corresponding FFT images are obtained from the grains in Fig. S2**g**. (**i**) TEM image of Pt nanomembrane and corresponding elements maps of Pt, C, and O

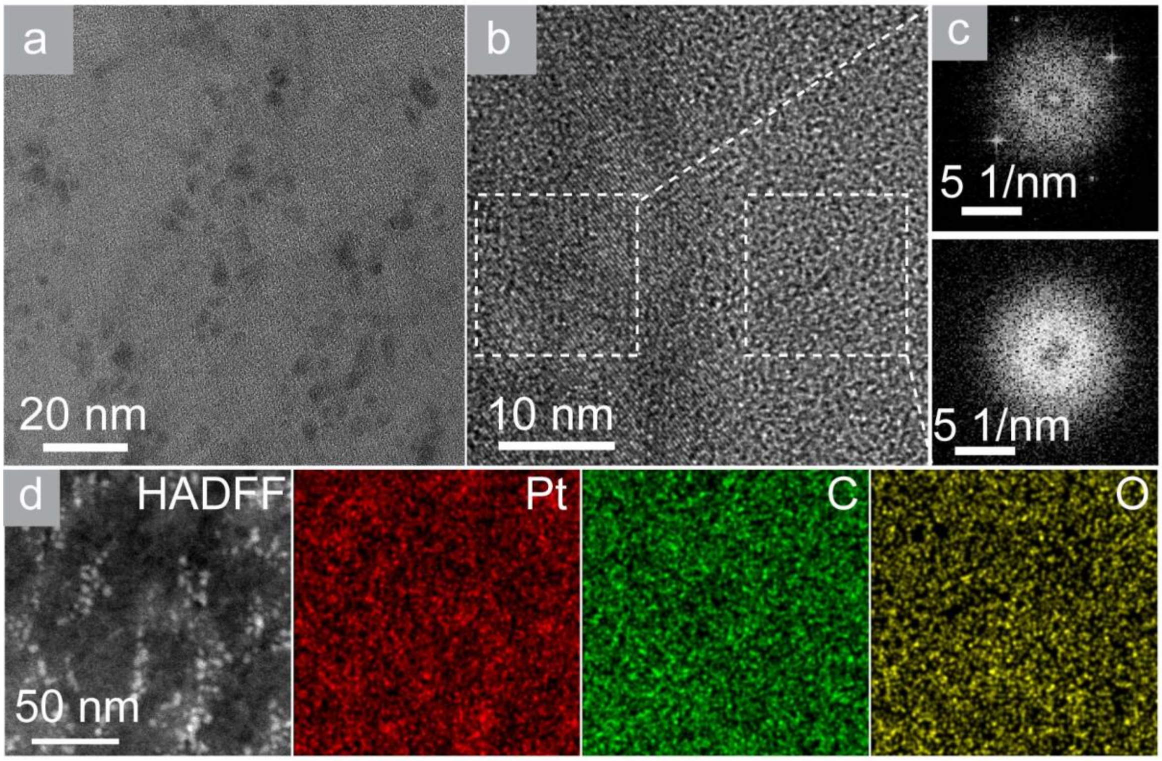

Fig. S4 TEM analysis of 5 nm Pt nanomembrane. (**a**) Low magnification of TEM image. (**b**) Enlarged TEM image, showing Pt nanoparticle and amorphous carbon. (**c**) The corresponding FFT images of (**b**). (**d**) HAADF-STEM image and corresponding elements maps of Pt, C and O

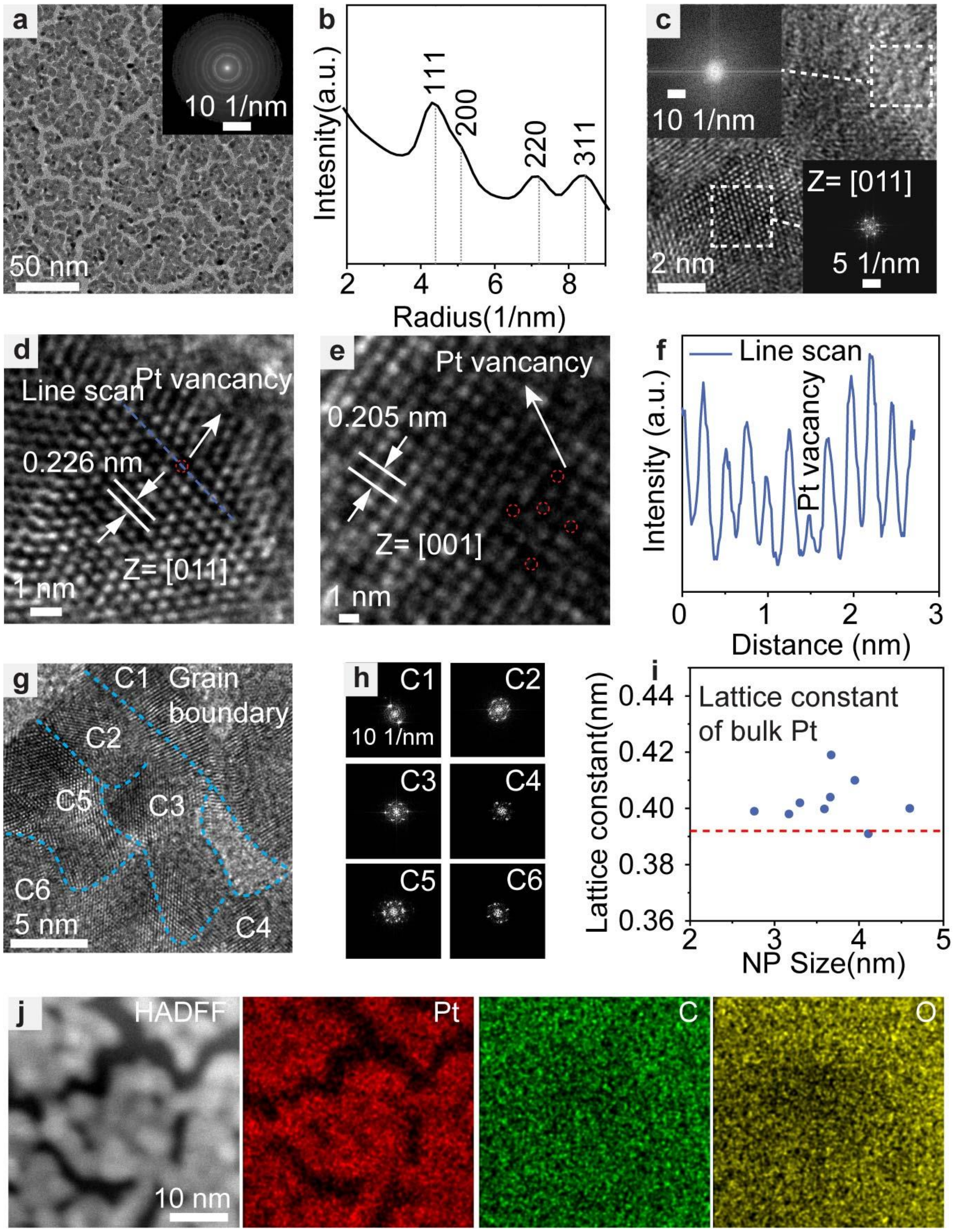

Fig. S5 TEM analysis of 10 nm Pt nanomembrane. (**a**) Low-magnification of TEM image, inset shows the corresponding SADP. (**b**) Radially integrated intensity of the diffraction patterns of the freestanding nanomembrane, in comparison with those of the single-phase FCC bulk Pt as indicated by the dash lines. (**c**) The high revolution TEM image of the 10 nm thick Pt nanomembrane. Insets are Fast Fourier transform (FFT) patterns of the amorphous (upper left) and crystalline (lower right) regions. (**d**, **e**) High resolution TEM images, showing the defects of vacancy in the orientation of [011] and [001] respectively. (**f**) Line scan of Intensity, showing the location of Pt vacancy. (**g**) Representative high-resolution TEM images of the Pt NC, the dashed line points out the grain boundaries. (**h**) The corresponding FFT images are obtained from the grains in Fig. S3**g**. (**i**) Lattice constant with the size of Pt nanocrystal (NC). (**j**) HAADF-STEM image and corresponding elements maps of Pt, C and O

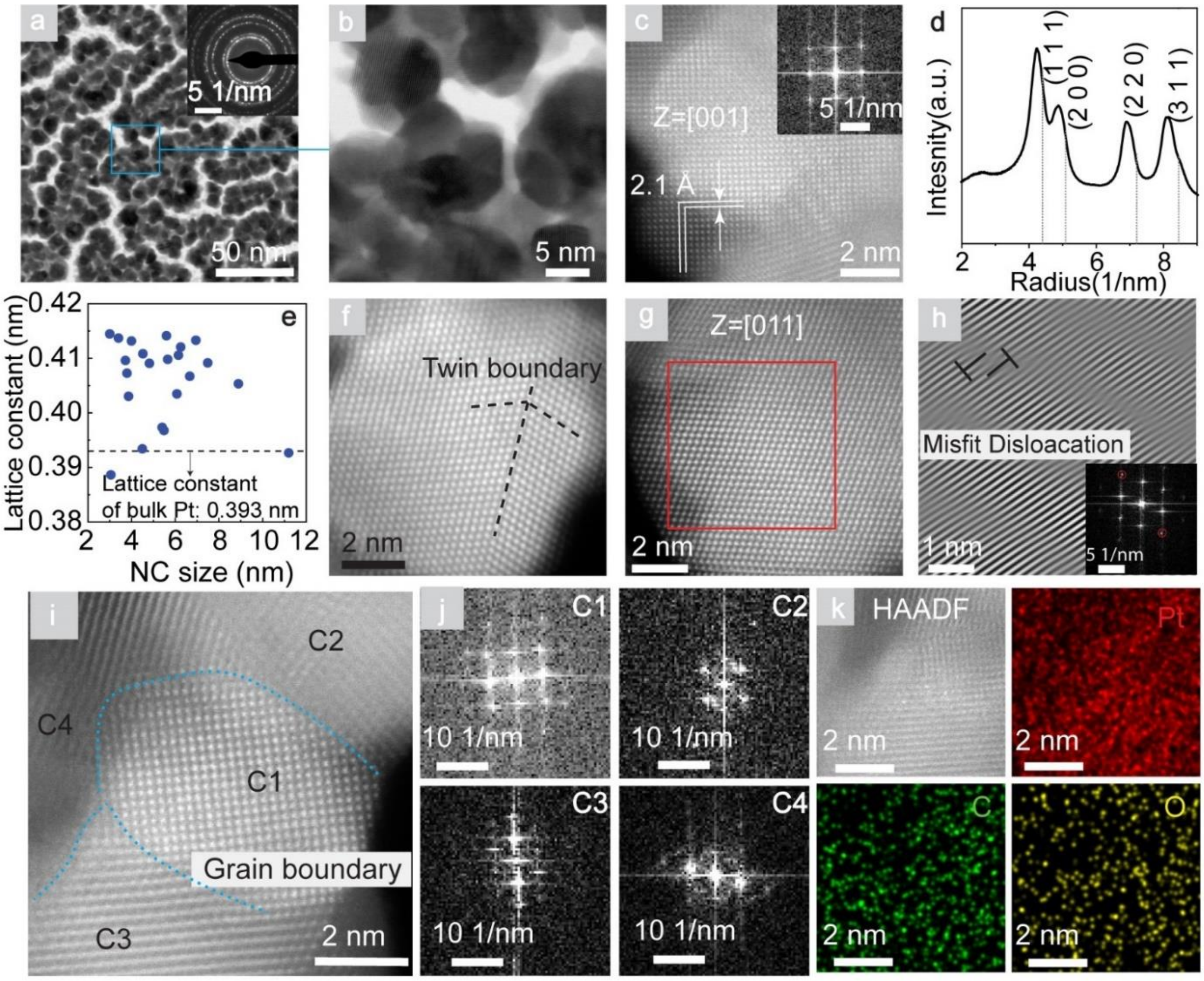

Fig. S6 TEM analysis of 28 nm Pt nanomembrane. (**a**) Low-magnification of HAADF-STEM image, inset is the corresponding SADP. (**b**) High magnification HAADF-STEM image of the 28 nm-thick freestanding Pt nanomembrane. (**c**) HAADF-STEM images from the [001] direction of a typical platinum NC with the FCC structure. (**d**) Radially integrated intensity of the diffraction patterns of the freestanding nanomembrane, in comparison with those of the single-phase FCC bulk Pt as indicated by the dash lines. (**e**) Lattice constant with the size of Pt NC. (**f**) Twin boundary. (**g**) HAADF-STEM images from the [011] direction of a typical Pt NC with a FCC structure. (**h**) The filtered IFFT image of the region in Fig. S4g, indicating misfit dislocation. (**i**) Representative high-resolution STEM images of the Pt NC, the dashed line points out the grain boundaries. (**j**) The corresponding FFT images are obtained from the grains in Fig. S4**i**. (**k**) HAADF-STEM image and corresponding elements maps of Pt, C and O

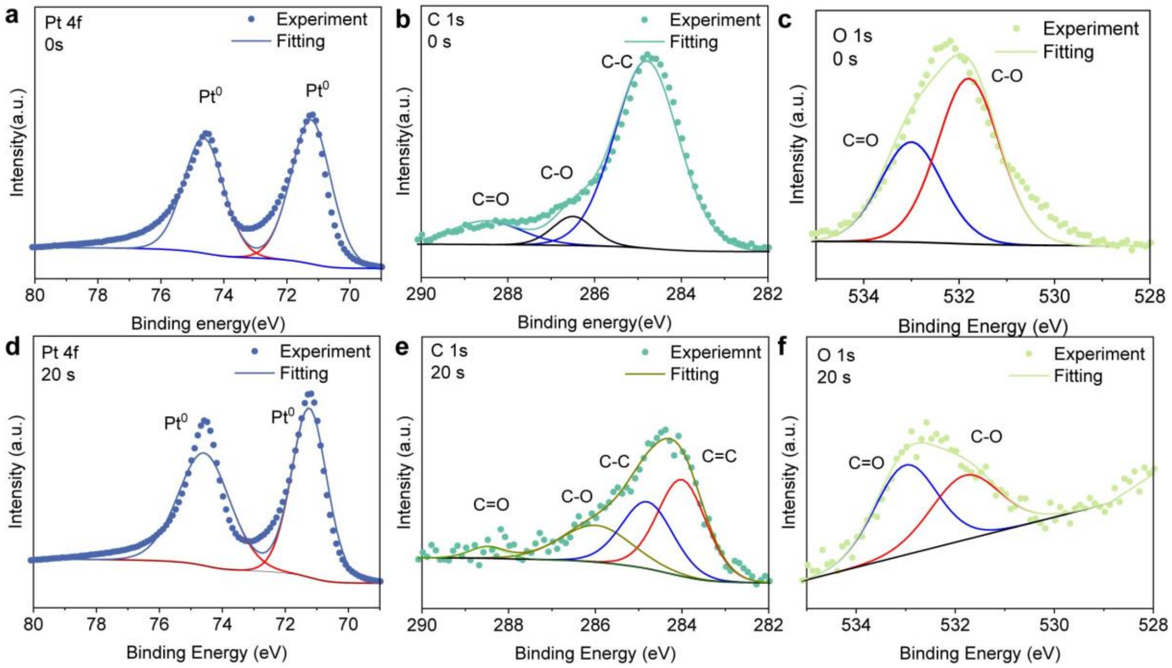

Fig. S7 X-ray photoelectron spectroscopy (XPS) narrow scanning with curve fitting based on the peaks of the constituent elements in the 19 nm Pt Nanomembrane. (**a-c**) were obtained at 0 s etching, (**d-f**) were obtained at 20 s etching

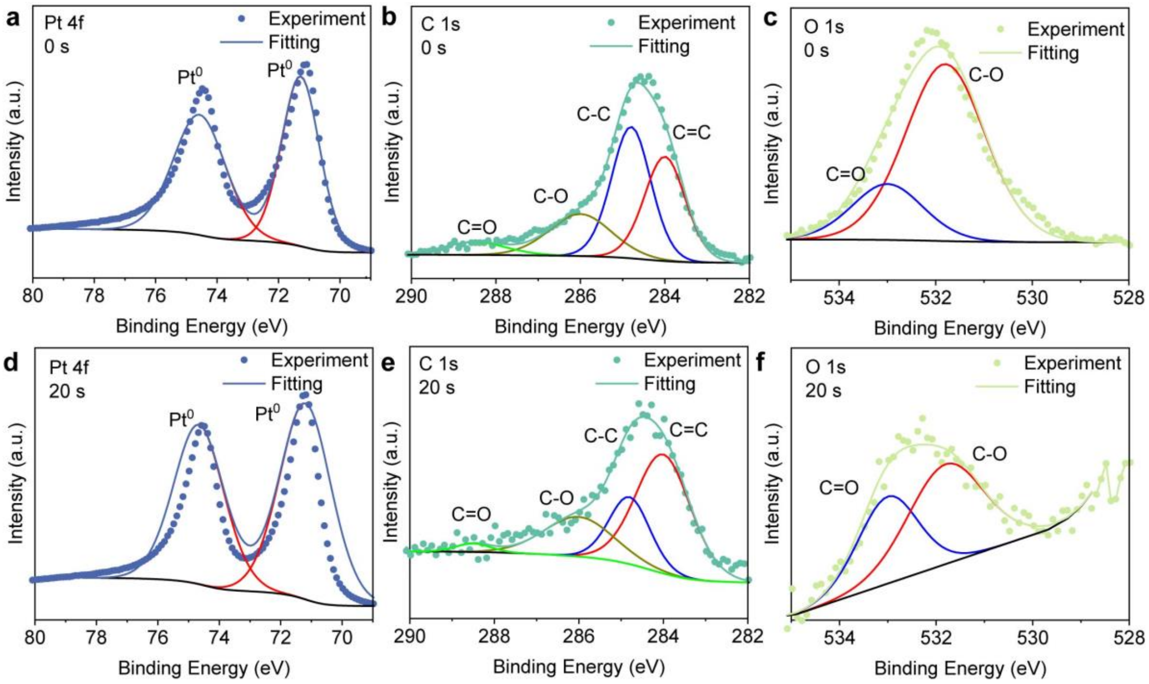

Fig. S8 X-ray photoelectron spectroscopy (XPS) narrow scanning with curve fitting based on the peaks of the constituent elements in the 28 nm Pt Nanomembrane. (**a-c**) were obtained at 0 s etching, (**d-f**) were obtained at 20 s etching

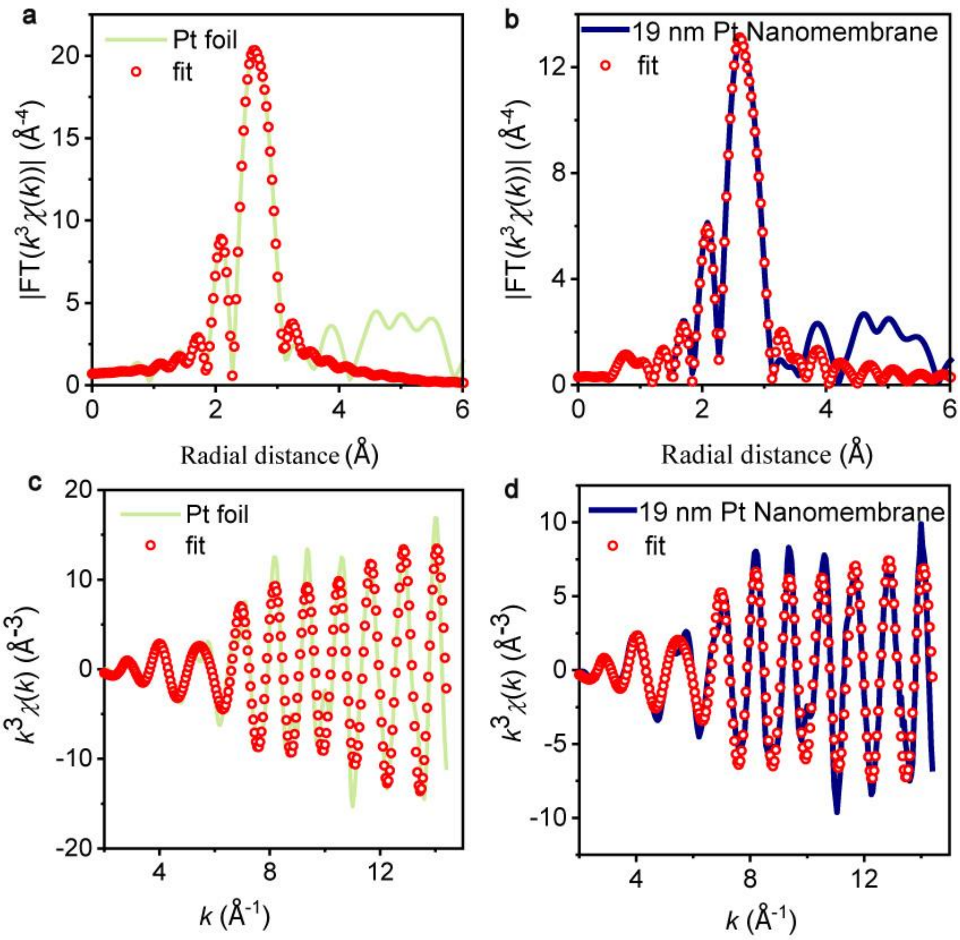

Fig. S9 EXAFS fitting results of Pt foil and 19 nm Pt nanomembrane. (**a**)-(**b**), FT-EXAFS spectra in r-space and the corresponding least-squares fit for the first shell of Pt foil and 19 nm Pt nanomembrane. (**c**)-(**d**), EXAFS χ(k) signals in k-space and the corresponding least-squares fit for Pt foil and 19 nm Pt nanomembrane

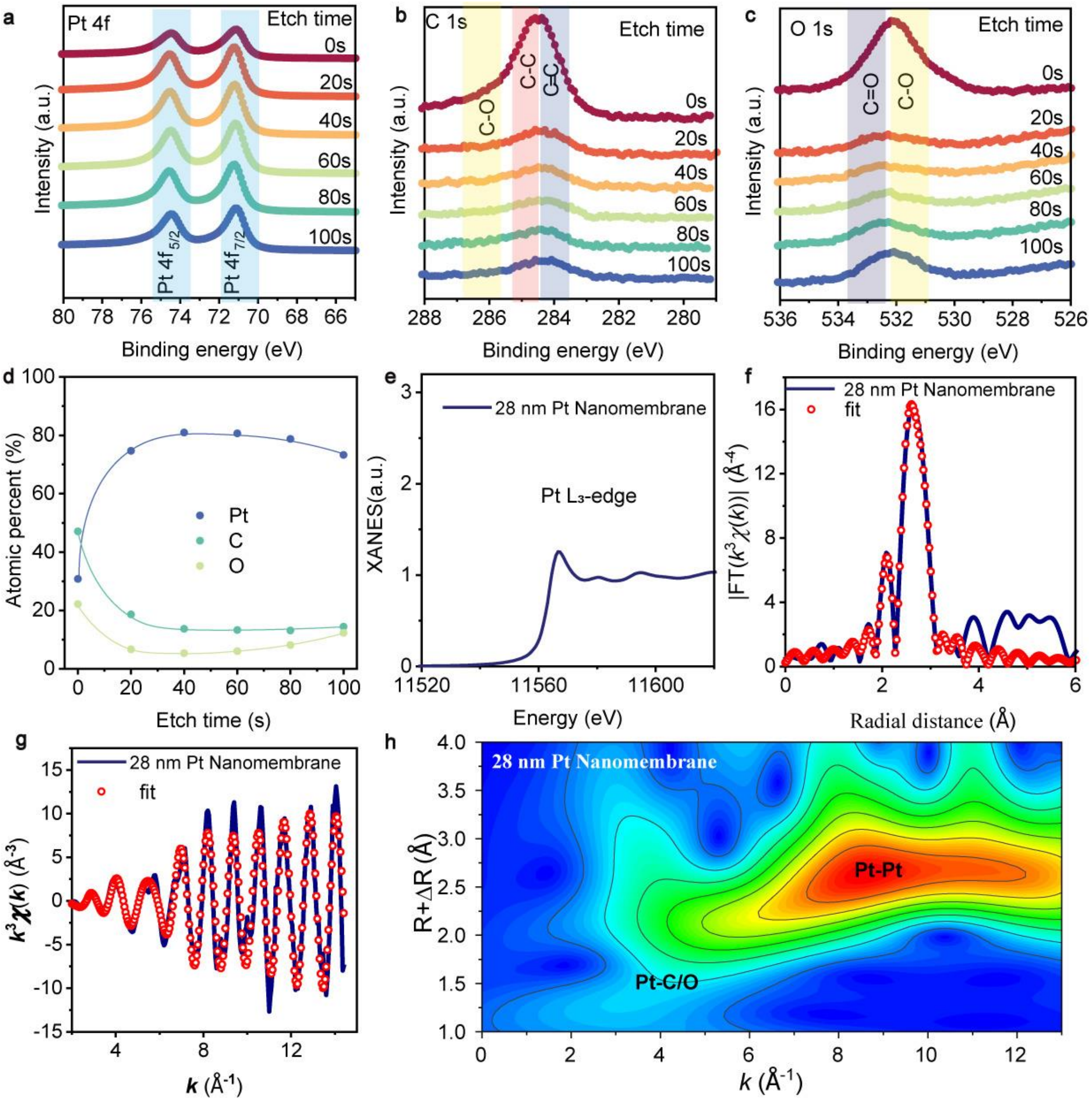

Fig. S10 Chemical bonding and atomic packing analyses of a 28 nm thick Pt nanomembrane. (**a**−**c**) Narrow-scan X-ray photoelectron spectroscopy (XPS) spectra for (a) Pt 4f, (**b**) C 1s, and (**c**) O 1s with the etching time. (**d**) Relative atomic concentration of Pt, C, and O with depth, as obtained from the quantitative analysis of the XPS data. (**e**) X-ray absorption near edge structure (XANES) at Pt L_3 edge. (**f**) FT-EXAFS region for the local structure of Pt and the corresponding least-squares fit for the first shell. (**g**) EXAFS χ(k) signals in *k*-space and the corresponding least-squares fit. (**h**) shows the wavelet transform for the *k*3-weighted EXAFS Pt K-edge signal of 28 nm Pt nanomembrane

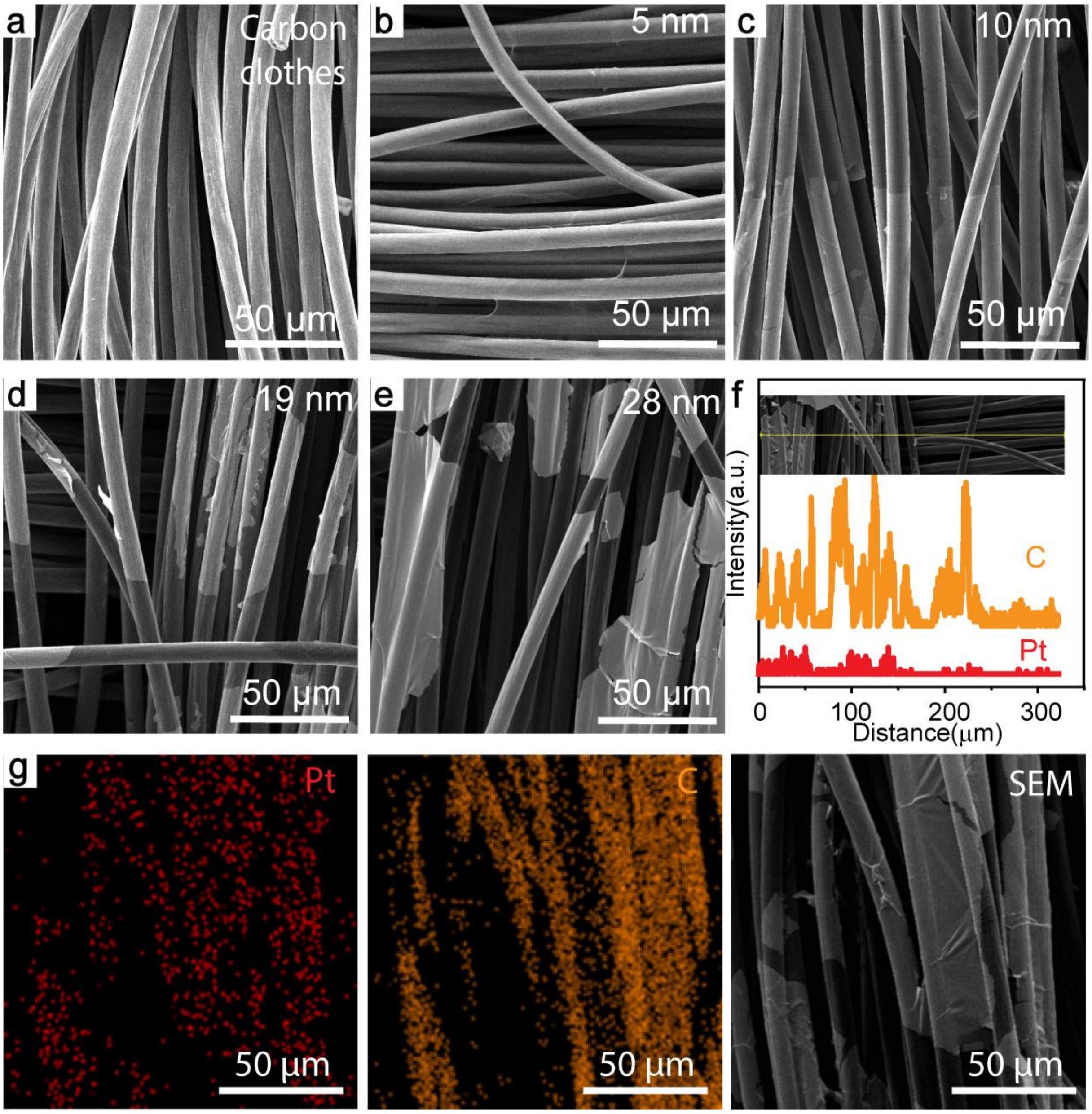

Fig. S11 Morphology and composition of the freestanding Pt nanomembrane transferred to the carbon clothes. (**a**) Scanning Electron Microscopy (SEM) image of Pure carbon clothes without Pt nanomembrane. (**b-e**) SEM images of 5 nm, 10 nm, 19 nm and 28 nm Pt nanomembrane transferred to carbon clothes. (**f**) Line scan of carbon clothes with 19 nm nanomembrane. (**g**) Energy Dispersive Spectroscopy (EDS) mapping of carbon clothes with 19 nm nanomembrane

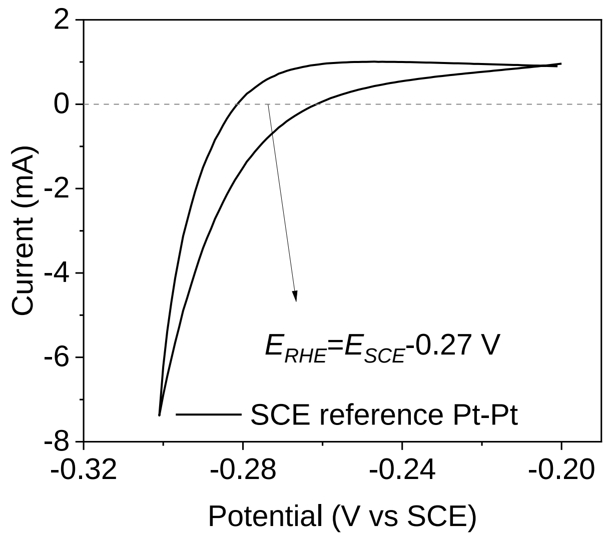

Fig. S12 Calibration of the saturated calomel electrode in 0.5 M H_2SO_4 with an H_2 atmosphere

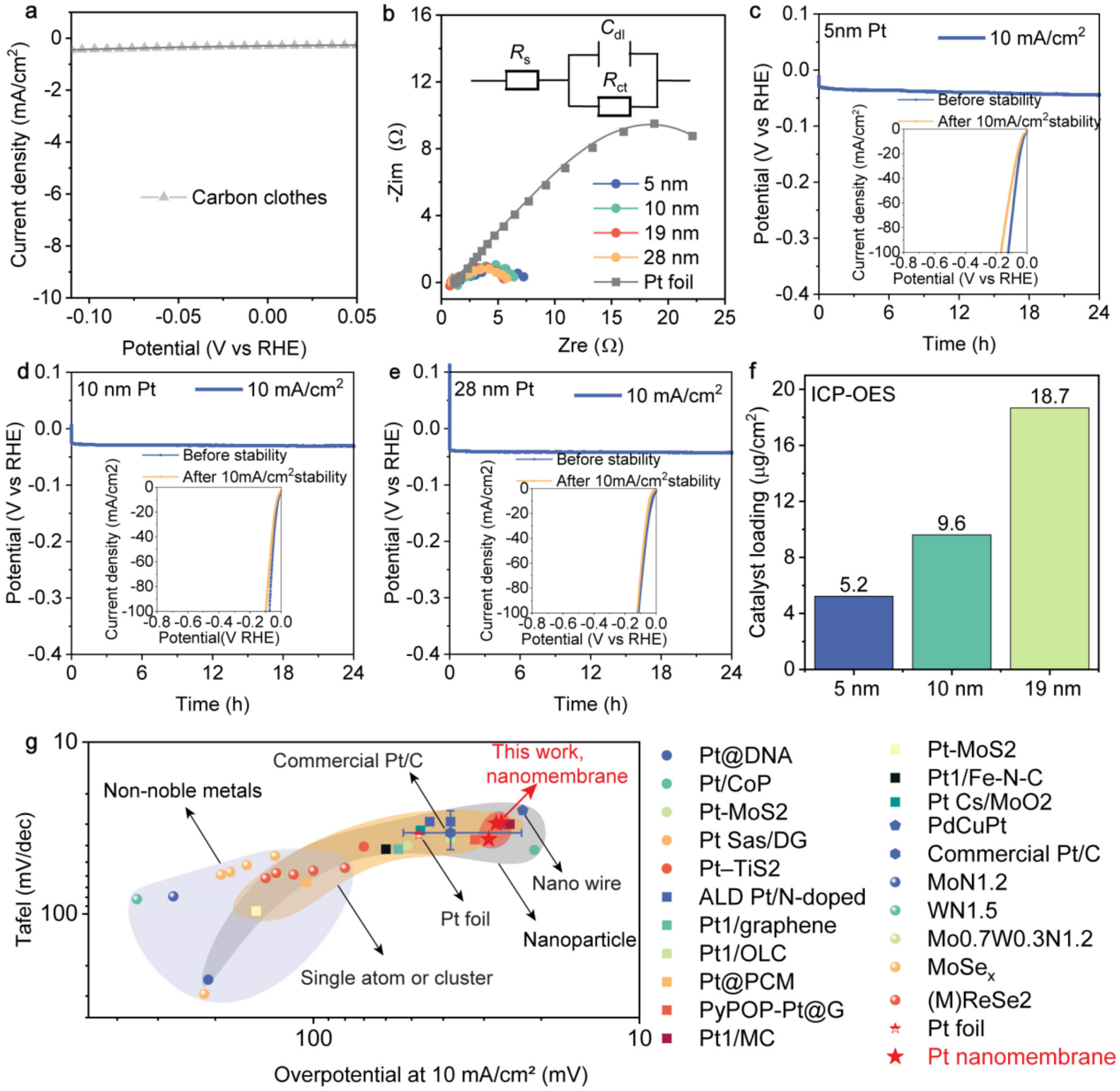

Fig. S13 (**a**) Linear Sweep Voltammetry (LSV) curve of Pure carbon clothes. (**b**) Electrochemical Impedance Spectrum (EIS) results of Pt nanomembrane and Pt foil at constant

potential -0.01 V vs RHE. (**c**)-(**e**) Stability tests of 5 nm, 10 nm and 28 nm Pt nanomembrane at 10 mA/cm^2 current density respectively, insets show LSV before and after stability tests. (**f**) Pt loading of our nanomembrane with different thicknesses measured by Inductively Coupled Plasma Optical Emission spectroscopy (ICP-OES). (**g**) Comparison of overpotential at 10 mA/cm^2 and Tafel slope with many other recently reported HER electrocatalysts, data from Table S2

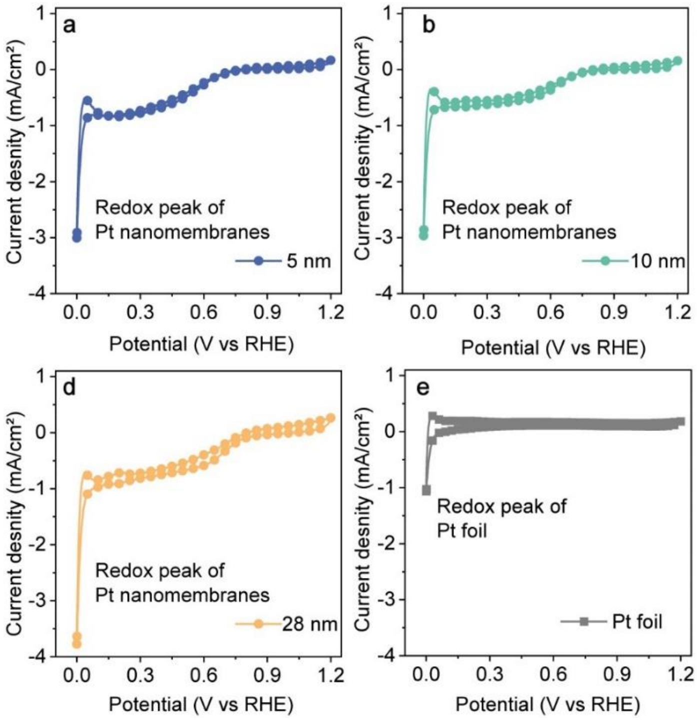

Fig. S14 Cyclic Voltammetry (CV) curves of (**a**) 5 nm, (**b**) 10 nm, (**c**) 19 nm, (**d**) 28 nm and (**e**) Pt foil at a scan rate of 50 mV/s

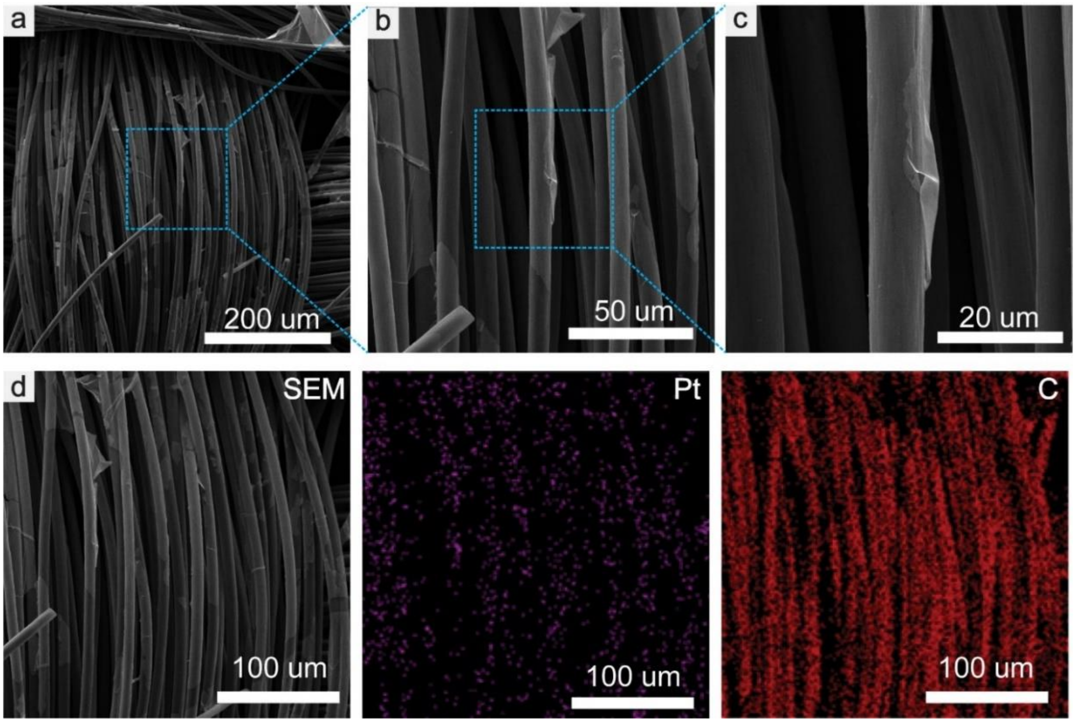

Fig. S15 (**a-c**) SEM images of 19 nm Pt nanomembrane transferred to carbon clothes after 24 hours 10 mA/cm^2 stability. (**d**) Energy Dispersive Spectroscopy (EDS) mapping of carbon clothes with 19 nm nanomembrane after stability tests

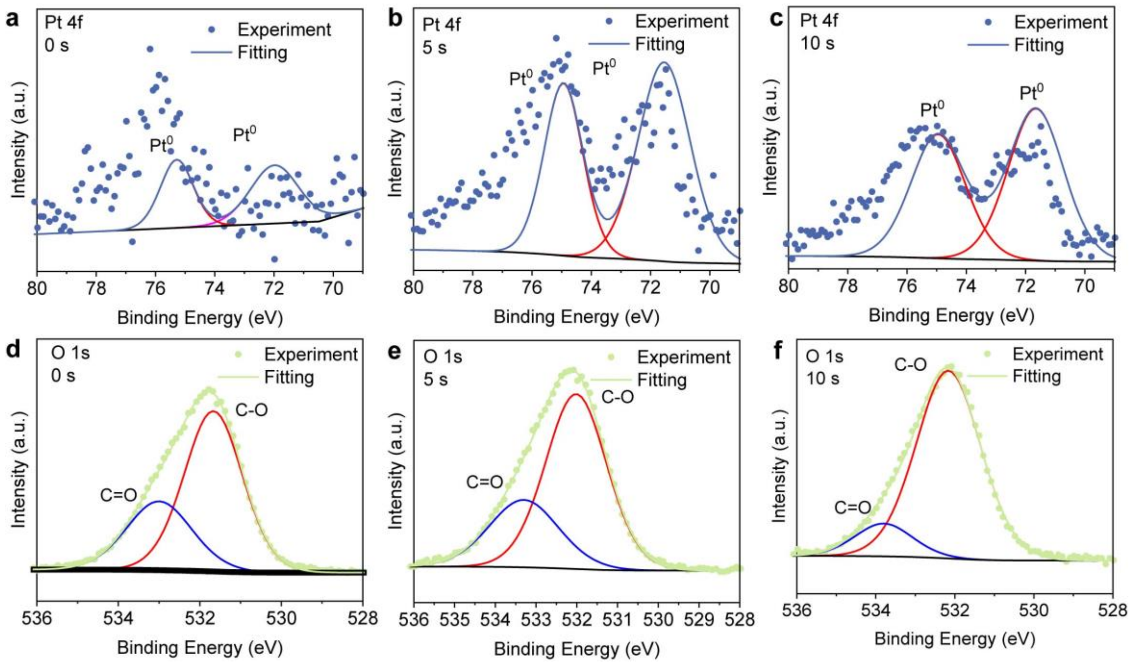

Fig. S16 X-ray photoelectron spectroscopy (XPS) depth profile analysis of Pt nanomembrane after stability tests. Narrow-scan XPS spectra for (**a-c**) Pt 4f and (**d-f**) O 1s as a function of etching time

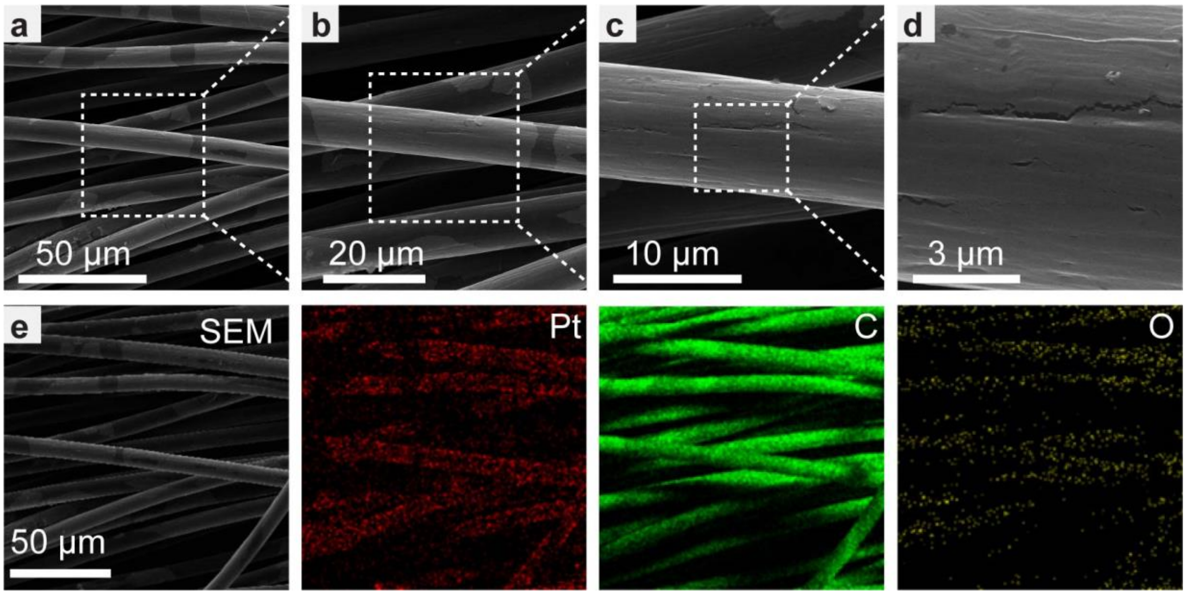

Fig. S17 Carbon clothes after stability tests and ultrasonic oscillation, (**a-d**) SEM images (**e**) SEM-EDS mapping Pt, C and O

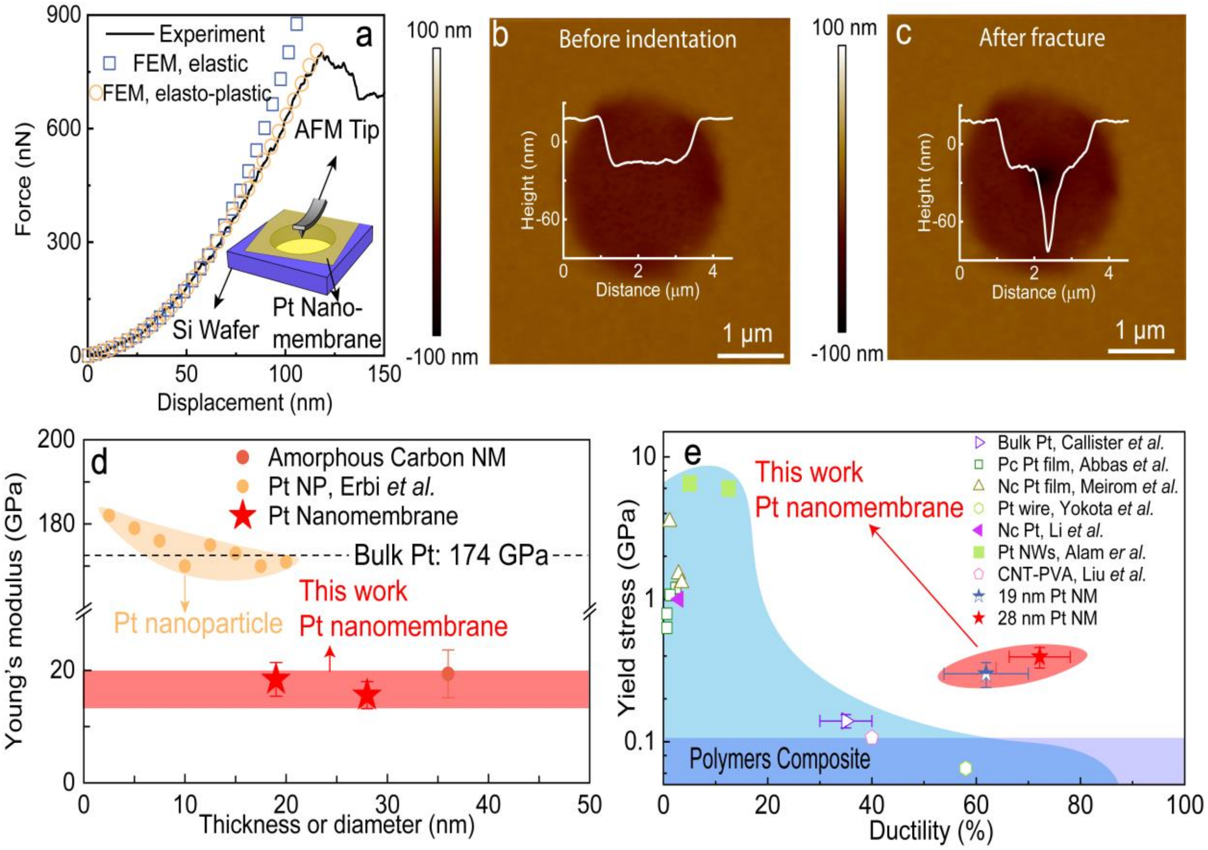

Fig. S18 Mechanical behavior of the freestanding Pt nanomembrane. (**a**) The experimental force−displacement curve of a 19 nm-thick Pt nanomembrane under AFM indentation in comparison with the FEA simulations. (**b**) and (**c**) AFM scanning images of the suspended Pt nanomembrane before and after indentation. (**d**) Comparison of Young's modulus of Pt nanomembrane, Pt nanoparticle (NP) [S1] and bulk Pt [S2]. (**e**) Comparison of the yield strength and ductility of Pt nanomembrane with those of nanocrystalline (NC) and polycrystalline (PC) Pt films, bulk Pt and Pt nanowires (NWs) [S3–S11]

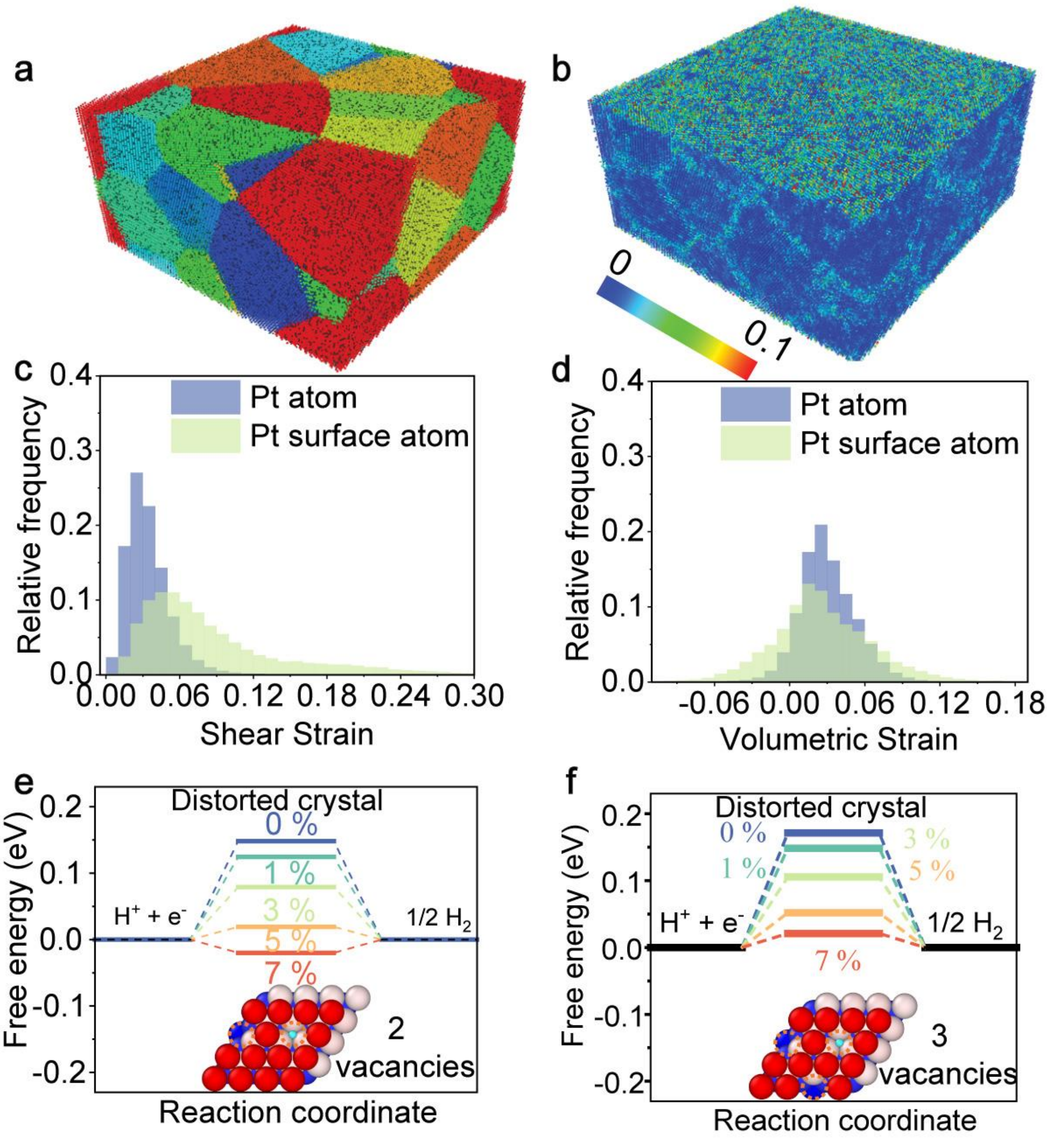

Fig. S19 Simulation of lattice distortion induced by substitutional defects and adsorption energy of distorted crystal. (**a**) Atomic configurations of Pt nanocrystalline with substitutional C atoms. The grain index is indicated by rainbow color coding where C atoms are colored by black. (**b**) Distribution of local shear strain which reflected by color coding. (**c**)-(**d**) Distribution of Shear Strain (**c**), Volumetric Strain (**d**) via Pt nanocrystalline with/without surface atoms, respectively. (**e**) Adsorption free energy versus the reaction coordinate of HER for a distorted crystal with 2 vacancies, with the heterogeneous tensile strain ranging from 0 to 7%. (**f**) Adsorption free energy versus the reaction coordinate of HER for a distorted crystal with 3 vacancies, with the heterogeneous tensile strain of 0–7%

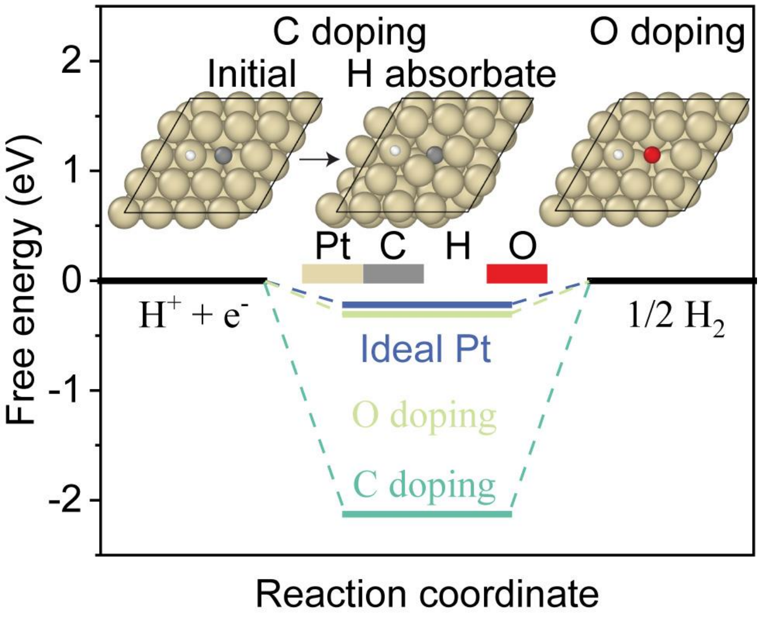

Fig. S20 Simulation of substitutional defects C or O, and adsorption free energy of a non-distorted Pt nanocrystal

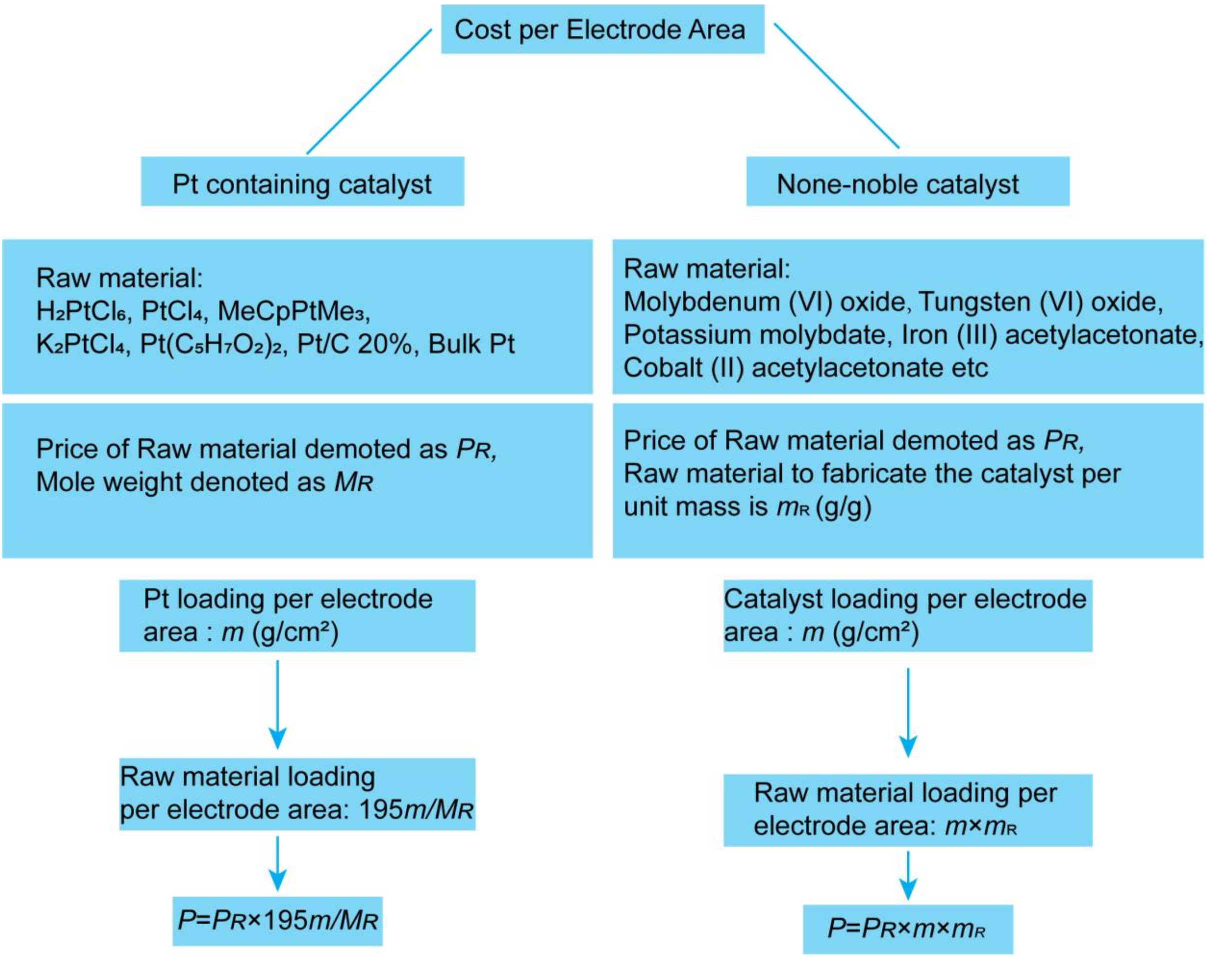

Fig. S21 Diagram illustrating how cost per electrode area is calculated based on the price of raw materials

Table S1 EXAFS fitting parameters at the Pt L_3-edge for Pt foil and Pt nanomembrane (S_0^2=0.839)

Sample	Shell	CN^a	R(Å)^b	σ^2(Å^2)^c	ΔE_0(eV)^d	R factor
Pt foil	Pt-Pt	12*	2.762±0.001	0.0048±0.0001	7.4±0.4	0.0017
**19 nm Pt nanomembrane**	**Pt-C/O**	**1.2±0.2**	**2.097±0.014**	**0.0092±0.0050**	**9.4±4.8**	**0.0074**
	**Pt-Pt**	**9.8±0.3**	**2.772±0.002**	**0.0061±0.0009**	**9.5±0.8**	
**28 nm Pt nanomembrane**	**Pt-C/O**	**0.8±0.2**	**2.101±0.023**	**0.0100±0.0026**	**4.1±2.6**	**0.0032**
	**Pt-Pt**	**10.2±0.4**	**2.779±0.002**	**0.0052±0.0006**	**10.6±0.5**	

$^a CN$, coordination number; $^b R$, the distance to the neighboring atom; $^c\sigma^2$, the Mean Square Relative Displacement (MSRD); $^d\Delta E_0$, inner potential correction; R factor indicates the goodness of the fit. S_0^2 was fixed to 0.839, according to the experimental EXAFS fit of Pt foil by fixing CN as the known crystallographic value. * This value was fixed during EXAFS fitting, based on the known structure of Pt. Fitting range: $3.0 \leq k$ (/Å) ≤ 13.8 and $1.0 \leq R$ (Å) ≤ 3.0 (Pt foil); $3.0 \leq k$ (/Å) ≤ 13.8 and $1.0 \leq R$ (Å) ≤ 3.5 (19 nm Pt nanomembrane); $3.0 \leq k$ (/Å) ≤ 13.8 and $1.0 \leq R$ (Å) ≤ 3.5 (28 nm Pt nanomembrane). A reasonable range of EXAFS fitting parameters: $0.700 < S_0^2 < 1.000$; $CN > 0$; σ2 > 0 Å2; $|\Delta E_0| < 10$ eV; R factor < 0.02.

Table S2 The comparison of HER performance of Pt nanomembrane with recently reported electrocatalysts in acid media

Catalysts	Loading (μg/cm^2)	η10 (mV)	Tafel (mV/dec)	Mass activity at 50 mV (A/mg)	Raw material	References
Pt-GT-1	1.4	18	N/A	21.43	H_2PtCl_6	[S12]
$Pd_{60}Pt_{40}$	13	130	N/A	0.15	$PtCl_4$	[S13]
Pt@DNA without binder	15	26	30	1.6	H_2PtCl_6	[S14]
Pt@DNA with binder	15	210	241	0.09	H_2PtCl_6	[S14]
Pt/CoP	102	21	42.5	0.38	H_2PtCl_6	[S15]
Pt-MoS_2	27	51.5	40	0.34	K_2PtCl_4	[S16]
Pt Sas/DG	1000	23	25	0.07	Pt net	[S17]
Pt–TiS_2	1.69	70	40.6	5.33	K_2PtCl_4	[S18]
ALD100Pt/N	6.1	44	29	10.1	$MeCpPtMe_3$	[S19]
ALD50Pt/N	1.61	38	29	2.12	$MeCpPtMe_3$	[S19]
Pt-ALD FTO	3.5	46.5	N/A	4	$MeCpPtMe_3$	[S20]
Pt1/graphene	1.74	55	42	4.77	$MeCpPtMe_3$	[S21]
Pt1/OLC	1.35	38	36	17.02	$MeCpPtMe_3$	[S21]
ALD Pt–WC	0.55	438	N/A	N/A	$MeCpPtMe_3$	[S22]
ALD Pt–WC	3.1	408	N/A	N/A	$MeCpPtMe_3$	[S22]
ALD Pt–WC	5.5	306	N/A	N/A	$MeCpPtMe_3$	[S22]
Pt@PCM	200	105	65.3	0.16	H_2PtCl_6	[S23]
PyPOP-Pt@G	67	32	37	0.84	H_2PtCl_6	[S24]
Pt1/MC	10	25	30	5.75	H_2PtCl_6	[S25]

Pt-MoS_2	18	150	96	0.09	H_2PtCl_6	[S26]
Pt1/Fe-N-C	300	60	42	0.02	H_2PtCl_6	[S27]
Pt Cs/MoO_2	1.75	47	32.6	7.43	H_2PtCl_6	[S28]
Pt_3Ni_3 NWS/C	15.3	31	N/A	N/A	$Pt(C_5H_7O_2)_2$	[S29]
PdCuPt	41	22.8	25	3.0	K_2PtCl_4	[S30]
40% Pt/C	640	63	32	0.013	N/A	[S26]
Pt/C	56.8	25.5	N/A	N/A	N/A	[S12]
Pt/C	33	52.5	31	0.27	N/A	[S19]
Pt/C	27	53	30	0.32	N/A	[S16]
Pt/C	80	14	32	N/A	N/A	[S31]
Pt/C	67.8	35	31.2	0.28	N/A	[S28]
Pt/C	53	32	34.1	0.37	N/A	[S32]
Pt/C without binder	20	41	33	0.72	N/A	[S14]
Pt/C with binder	20	56	60	0.4	N/A	[S14]
Pt/C 20%	95.2	29	35	0.27	N/A	[S21]
Pt/C 5%	25.9	50	40	0.38	N/A	[S21]
Pt/C	59.4	15	21	N/A	N/A	[S33]
Pt/C	36.1	30	31	0.83	N/A	[S17]
Pt/C	41.5	25	28	1.325	N/A	[S30]
$MoN_{1.2}$	400	269	79	N/A	N/A	[S34]
$WN_{1.5}$	400	348	82	N/A	N/A	[S34]
$Mo_{0.7}W_{0.3}N_{1.2}$	400	129	59	N/A	N/A	[S34]
$MoSe_{1.8}$	390	216	292	N/A	N/A	[S35]
$MoSe_{2.0}$	390	192	59	N/A	N/A	[S35]
$MoSe_{2.2}$	390	160	52	N/A	N/A	[S35]
$MoSe_{2.3}$	390	131	46	N/A	N/A	[S35]
$MoSe_{2.4}$	390	180	57	N/A	N/A	[S35]
$ReSe_2$	390	140	62	N/A	N/A	[S36]
$MnReSe_2$	390	140	62	N/A	N/A	[S36]
$FeReSe_2$	390	115	59	N/A	N/A	[S36]
$CoReSe_2$	390	100	56	N/A	N/A	[S36]
$NiReSe_2$	390	80	54	N/A	N/A	[S36]
$CuReSe_2$	390	130	58	N/A	N/A	[S36]
**Pt foil**	**321750**	**47.5**	**34.5**	**5.7E-5**	**Pure Pt**	**This work**
**5 nm Pt nanomembrane**	**5.22**	**29**	**36.7**	**4.43**	**Pure Pt**	**This work**
**10 nm Pt nanomembrane**	**9.6**	**26.5**	**29.2**	**2.67**	**Pure Pt**	**This work**
**19 nm Pt nanomembrane**	**18.6**	**27.5**	**29.7**	**1.38**	**Pure Pt**	**This work**

Table S3 Summary of the TOF values and current densities at the overpotential of 100 mV between Our Pt nanomembrane and many other Pt-based catalysts in 0.5 M H_2SO_4

Catalysts	Current density at 100 mV (mA/cm^2)	TOF 100mV(s^{-1})	References
PtGa	500	17.1	[S37]
Pt-Ru/CNT	265	25.1	[S38]
Pt1 SAC-VNGNMA	88	4.0	[S39]
Pt1/OLC	56.5	40.8	[S21]
Pt-SAs/WS_2	54	131.7	[S40]
Pt_2W/WO_3/RGO	44	2	[S41]
Pt1/NMHCS	40	4.5	[S42]
PtRu@RFCS-6h	32	4.0	[S43]
Pt-SAs/MoS_2	24	47.3	[S44]
Pt@PCM	11	3.2	[S23]
**5 nm Pt nanomembrane**	**74.7**	**145.7**	**This Work**
**10 nm Pt nanomembrane**	**83.8**	**90.2**	**This Work**
**19 nm Pt nanomembrane**	**89.7**	**48.3**	**This Work**

Table S4 Price of raw materials for noble catalysts

Raw material	Price per mass ($/g)	Molecular Weight	Pt Price per mass ($/μg)	References
H_2PtCl_6	270.71	409.81	0.13	[S45]
$PtCl_4$	178.00	336.89	0.10	[S46]
$MeCpPtMe_3$	475.76	319.30	0.29	[S47]
K_2PtCl_4	137.30	415.00	0.06	[S48]
$Pt(C_5H_7O_2)_2$	229.40	393.29	0.11	[S49]
Pt/C 20%	152.00	195.00	7.60E-04	[S50]
Bulk Pt	33.86	195.00	3.39E-05	[S51]

Table S5 Price of raw material for non-noble catalysts

Raw material	Price per gram ($/g)	References
Molybdenum (VI) oxide	2.87	[S52]
Tungsten (VI) oxide	15.48	[S53]
Potassium molybdate	0.94	[S54]
Ammonium perrhenate	22.68	[S55]
Selenium	9.59	[S56]
Dibenzyl diselenide, 95%, Thermo Scientific Chemicals	21.50	[S57]
Manganese (III) acetylacetonate	2.32	[S58]
Iron (III) acetylacetonate	0.11	[S59]
Cobalt (II) acetylacetonate	1.85	[S60]
Nickel (II) acetylacetonate	9.20	[S61]
Copper (II) acetylacetonate	4.70	[S62]

Supplementary References

[S1] M. Erbi, H. Amara, R. Gatti, Tuning elastic properties of metallic nanoparticles by shape controlling: from atomistic to continuous models.: arXiv: 2303.06995. (2023).

[S2] K.N. Jonnalagadda, I. Chasiotis, S. Yagnamurthy, J. Lambros, J. Pulskamp et al., Experimental investigation of strain rate dependence of nanocrystalline Pt films. Exp. Mech. **50**, 25–35 (2010). https://doi.org/10.1007/s11340-008-9212-7

[S3] R.A. Meirom, T.E. Clark, C.L. Muhlstein, The effects of texture and grain morphology on the fracture toughness and fatigue crack growth resistance of nanocrystalline platinum films. Int. J. Fatigue **70**, 258–269 (2015). https://doi.org/10.1016/j.ijfatigue.2014.09.017

[S4] R.A. Meirom, D.H. Alsem, A.L. Romasco, T. Clark, R.G. Polcawich et al., Fatigue-induced grain coarsening in nanocrystalline platinum films. Acta Mater. **59**, 1141–1149 (2011). https://doi.org/10.1016/j.actamat.2010.10.047

[S5] R.A. Meirom, T.E. Clark, C.L. Muhlstein, The role of specimen thickness in the fracture toughness and fatigue crack growth resistance of nanocrystalline platinum films. Acta Mater. **60**, 1408–1417 (2012). https://doi.org/10.1016/j.actamat.2011.11.015

[S6] K. Abbas, S. Alaie, M. Ghasemi Baboly, M.M. Elahi, D.H. Anjum et al., Nanoscale size effects on the mechanical properties of platinum thin films and cross-sectional grain morphology. J. Micromech. Microeng. **26**, 015007 (2016). https://doi.org/10.1088/0960-1317/26/1/015007

[S7] M.F. Alam, M.R. Bin Shahadat, Temperature and strain rate dependent mechanical properties of ultrathin metallic nanowires: A molecular dynamics study. AIP Conf. Proc. **1980**, 030015 (2018) https://doi.org/10.1063/1.5044294

[S8] J. Li, B. Lu, H. Zhou, C. Tian, Y. Xian et al., Molecular dynamics simulation of mechanical properties of nanocrystalline platinum: grain-size and temperature effects. Phys. Lett. A **383**, 1922–1928 (2019). https://doi.org/10.1016/j.physleta.2018.10.053

[S9] Y. Yokota, T. Nihei, M. Yoshino, A. Yamaji, S. Toyoda et al., Microstructure and mechanical properties of platinum fiber fabricated by unidirectional solidification. Crystals **10**, 216 (2020). https://doi.org/10.3390/cryst10030216

[S10] W. D. Callister Jr and D. G. Rethwisch, *Fundamentals of materials science and engineering: an integrated approach*. Fundamentals of Materials Science and Engineering: An Integrated Approach (John Wiley \& Sons, 2020)

[S11] L. Liu, A.H. Barber, S. Nuriel, H.D. Wagner, Mechanical properties of functionalized single-walled carbon-nanotube/poly(vinyl alcohol) nanocomposites. Adv. Funct. Mater. **15**, 975–980 (2005). https://doi.org/10.1002/adfm.200400525

[S12] J.N. Tiwari, S. Sultan, C.W. Myung, T. Yoon, N. Li et al., Multicomponent electrocatalyst with ultralow Pt loading and high hydrogen evolution activity. Nat. Energy **3**, 773–782 (2018). https://doi.org/10.1038/s41560-018-0209-x

[S13] R. Ojani, J.B. Raoof, E. Hasheminejad, One-step electroless deposition of Pd/Pt bimetallic microstructures by galvanic replacement on copper substrate and investigation of its performance for the hydrogen evolution reaction. Int. J. Hydrog. Energy **38**, 92–99 (2013). https://doi.org/10.1016/j.ijhydene.2012.10.015

[S14] S. Anantharaj, P.E. Karthik, B. Subramanian, S. Kundu, Pt nanoparticle anchored molecular self-assemblies of DNA: an extremely stable and efficient HER electrocatalyst with ultralow Pt content. ACS Catal. **6**, 4660–4672 (2016). https://doi.org/10.1021/acscatal.6b00965

[S15] J. Li, H.-X. Liu, W. Gou, M. Zhang, Z. Xia et al., Ethylene-glycol ligand environment facilitates highly efficient hydrogen evolution of Pt/CoP through proton concentration and hydrogen spillover. Energy Environ. Sci. **12**, 2298–2304 (2019). https://doi.org/10.1039/C9EE00752K

[S16] X. Huang, Z. Zeng, S. Bao, M. Wang, X. Qi et al., Solution-phase epitaxial growth of noble metal nanostructures on dispersible single-layer molybdenum disulfide nanosheets. Nat. Commun. **4**, 1444 (2013). https://doi.org/10.1038/ncomms2472

[S17] Y. Qu, B. Chen, Z. Li, X. Duan, L. Wang et al., Thermal emitting strategy to synthesize atomically dispersed Pt metal sites from bulk Pt metal. J. Am. Chem. Soc. **141**, 4505–4509 (2019). https://doi.org/10.1021/jacs.8b09834

[S18] Z. Zeng, C. Tan, X. Huang, S. Bao, H. Zhang, Growth of noble metal nanoparticles on single-layer TiS_2 and TaS_2nanosheets for hydrogen evolution reaction. Energy Environ. Sci. **7**, 797–803 (2014). https://doi.org/10.1039/c3ee42620c

[S19] N. Cheng, S. Stambula, D. Wang, M.N. Banis, J. Liu et al., Platinum single-atom and cluster catalysis of the hydrogen evolution reaction. Nat. Commun. **7**, 13638 (2016). https://doi.org/10.1038/ncomms13638

[S20] J. Dendooven, R.K. Ramachandran, E. Solano, M. Kurttepeli, L. Geerts et al., Independent tuning of size and coverage of supported Pt nanoparticles using atomic layer deposition. Nat. Commun. **8**, 1074 (2017). https://doi.org/10.1038/s41467-017-01140-z

[S21] D. Liu, X. Li, S. Chen, H. Yan, C. Wang et al., Atomically dispersed platinum supported on curved carbon supports for efficient electrocatalytic hydrogen evolution. Nat. Energy **4**, 512–518 (2019). https://doi.org/10.1038/s41560-019-0402-6

[S22] I.J. Hsu, Y.C. Kimmel, X. Jiang, B.G. Willis, J.G. Chen, Atomic layer deposition synthesis of platinum–tungsten carbide core–shell catalysts for the hydrogen evolution reaction. Chem. Commun. **48**, 1063–1065 (2012). https://doi.org/10.1039/C1CC15812K

[S23] H. Zhang, P. An, W. Zhou, B.Y. Guan, P. Zhang et al., Dynamic traction of lattice-confined platinum atoms into mesoporous carbon matrix for hydrogen evolution reaction. Sci. Adv. **4**, eaao6657 (2018). https://doi.org/10.1126/sciadv.aao6657

[S24] A.B. Soliman, M.H. Hassan, T.N. Huan, A.A. Abugable, W.A. Elmehalmey et al., Pt immobilization within a tailored porous-organic polymer–graphene composite: opportunities in the hydrogen evolving reaction. ACS Catal. **7**, 7847–7854 (2017). https://doi.org/10.1021/acscatal.7b02246

[S25] H. Wei, K. Huang, D. Wang, R. Zhang, B. Ge et al., Iced photochemical reduction to synthesize atomically dispersed metals by suppressing nanocrystal growth. Nat. Commun. **8**, 1490 (2017). https://doi.org/10.1038/s41467-017-01521-4

[S26] J. Deng, H. Li, J. Xiao, Y. Tu, D. Deng et al., Triggering the electrocatalytic hydrogen evolution activity of the inert two-dimensional MoS_2 surface *via* single-atom metal doping. Energy Environ. Sci. **8**, 1594–1601 (2015). https://doi.org/10.1039/C5EE00751H

[S27] X. Zeng, J. Shui, X. Liu, Q. Liu, Y. Li et al., Single-atom to single-atom grafting of Pt_1 onto Fe N$_4$ center: Pt_1@Fe-N-C multifunctional electrocatalyst with significantly enhanced properties. Adv. Energy Mater. **8**, 1701345 (2018). https://doi.org/10.1002/aenm.201701345

[S28] X. Li, J. Yu, J. Jia, A. Wang, L. Zhao et al., Confined distribution of platinum clusters on MoO_2 hexagonal nanosheets with oxygen vacancies as a high-efficiency electrocatalyst for hydrogen evolution reaction. Nano Energy **62**, 127–135 (2019). https://doi.org/10.1016/j.nanoen.2019.05.013

[S29] P. Wang, X. Zhang, J. Zhang, S. Wan, S. Guo et al., Precise tuning in platinum-nickel/nickel sulfide interface nanowires for synergistic hydrogen evolution catalysis. Nat. Commun. **8**, 14580 (2017). https://doi.org/10.1038/ncomms14580

[S30] T. Chao, X. Luo, W. Chen, B. Jiang, J. Ge et al., Atomically dispersed copper-platinum dual sites alloyed with palladium nanorings catalyze the hydrogen evolution reaction. Angew. Chem. Int. Ed Engl. **56**, 16047–16051 (2017). https://doi.org/10.1002/anie.201709803

[S31] H. Jin, S. Sultan, M. Ha, J.N. Tiwari, M.G. Kim et al., Simple and scalable mechanochemical synthesis of noble metal catalysts with single atoms toward highly efficient hydrogen evolution. Adv. Funct. Mater. **30**, 2000531 (2020). https://doi.org/10.1002/adfm.202000531

[S32] B. Lin, Y. Zhang, H. Zhang, H. Wu, J. Shao et al., Centimeter-scale two-dimensional metallenes for high-efficiency electrocatalysis and sensing. ACS Mater. Lett. **5**, 397–405 (2023). https://doi.org/10.1021/acsmaterialslett.2c01066

[S33] H. Jin, M. Ha, M.G. Kim, J.H. Lee, K.S. Kim, Engineering Pt coordination environment with atomically dispersed transition metal sites toward superior hydrogen evolution. Adv. Energy Mater. **13**, 2204213 (2023). https://doi.org/10.1002/aenm.202204213

[S34] H. Jin, Q. Gu, B. Chen, C. Tang, Y. Zheng et al., Molten salt-directed catalytic synthesis of 2D layered transition-metal nitrides for efficient hydrogen evolution. Chem **6**, 2382–2394 (2020). https://doi.org/10.1016/j.chempr.2020.06.037

[S35] I.S. Kwon, I.H. Kwak, T.T. Debela, H.G. Abbas, Y.C. Park et al., Se-rich $MoSe_2$ nanosheets and their superior electrocatalytic performance for hydrogen evolution reaction. ACS Nano **14**, 6295–6304 (2020). https://doi.org/10.1021/acsnano.0c02593

[S36] I.S. Kwon, I.H. Kwak, S. Ju, S. Kang, S. Han et al., Adatom doping of transition metals in $ReSe_2$ nanosheets for enhanced electrocatalytic hydrogen evolution reaction. ACS Nano **14**, 12184–12194 (2020). https://doi.org/10.1021/acsnano.0c05874

[S37] Q. Yang, G. Li, K. Manna, F. Fan, C. Felser et al., Topological engineering of Pt-group-metal-based chiral crystals toward high-efficiency hydrogen evolution catalysts. Adv. Mater. **32**, e1908518 (2020). https://doi.org/10.1002/adma.201908518

[S38] D. Zhang, Z. Wang, X. Wu, Y. Shi, N. Nie et al., Noble metal (Pt, Rh, Pd, Ir) doped Ru/CNT ultra-small alloy for acidic hydrogen evolution at high current density. Small **18**, e2104559 (2022). https://doi.org/10.1002/smll.202104559

[S39] K. Chi, Z. Chen, F. Xiao, W. Guo, W. Xi et al., Maximizing the utility of single atom electrocatalysts on a 3D graphene nanomesh. J. Mater. Chem. A **7**, 15575–15579 (2019). https://doi.org/10.1039/C9TA00942F

[S40] Y. Shi, Z.-R. Ma, Y.-Y. Xiao, Y.-C. Yin, W.-M. Huang et al., Electronic metal-support interaction modulates single-atom platinum catalysis for hydrogen evolution reaction. Nat. Commun. **12**, 3021 (2021). https://doi.org/10.1038/s41467-021-23306-6

[S41] Y.-W. Peng, C. Shan, H.-J. Wang, L. Hong, S. Yao et al., Polyoxometalate-derived ultrasmall Pt_2W/WO_3 heterostructure outperforms platinum for large-current-density H_2 evolution. Adv. Energy Mater. **9**, 1–9 (2019). https://doi.org/10.1002/aenm.201900597

[S42] P. Kuang, Y. Wang, B. Zhu, F. Xia, C.W. Tung et al., Pt single atoms supported on N-doped mesoporous hollow carbon spheres with enhanced electrocatalytic H_2-evolution activity. Adv. Mater. **33**, e2008599 (2021). https://doi.org/10.1002/adma.202008599

[S43] K. Li, Y. Li, Y. Wang, J. Ge, C. Liu et al., Enhanced electrocatalytic performance for the hydrogen evolution reaction through surface enrichment of platinum nanoclusters alloying with ruthenium *in situ* embedded in carbon. Energy Environ. Sci. **11**, 1232–1239 (2018). https://doi.org/10.1039/C8EE00402A

[S44] Y. Shi, W.-M. Huang, J. Li, Y. Zhou, Z.-Q. Li et al., Site-specific electrodeposition enables self-terminating growth of atomically dispersed metal catalysts. Nat. Commun. **11**, 4558 (2020). https://doi.org/10.1038/s41467-020-18430-8

[S45] Merck, Chloroplatinic acid solution. (2023). https://www.sigmaaldrich.com/HK/en/product/aldrich/262587?gclid=Cj0KCQjwk7ugB

hDIARIsAGuvgPYmy_nhnnZ3hdrqGp9ehvTpe0HVD8i2vMSK-0ZbjpV-XtvL-MRawM4aAmE5EALw_wcB

[S46] Merck, Platinum(IV) chloride. (2023). https://www.sigmaaldrich.com/HK/en/product/aldrich/206113

[S47] Merck, Trimethyl(methylcyclopentadienyl)platinum(IV). (2023). https://www.sigmaaldrich.com/HK/en/product/aldrich/645605

[S48] Merck, Potassium tetrachloroplatinate(II). (2023). https://www.sigmaaldrich.com/HK/en/product/aldrich/206075?gclid=Cj0KCQjwk7ugBhDIARIsAGuvgPZDGlxPjYs31bM2dkrOnXMxY-Kc6h2-yl5HusYx2B3j69T0RTo1nRUaAn9JEALw_wcB

[S49] Merck, Platinum(II) acetylacetonate. (2023). https://www.sigmaaldrich.com/HK/zh/product/aldrich/282782?gclid=Cj0KCQjwk7ugBhDIARIsAGuvgPZWez6vbLp2ImVg1mPPAjXtHJjwqMIhYvh3WxQLgrPsJ5oKi1d3JgUaAgUiEALw_wcB

[S50] Merk, Platinum on graphitized carbon. (2023). https://www.sigmaaldrich.com/HK/en/product/aldrich/738549

[S51] the New York Mercantile Exchange, Pt. (2023). https://www.cbcie.com/pt/index.html

[S52] Merck, Molybdenum(VI) oxide. (2023). https://www.sigmaaldrich.com/HK/en/search/moo3?focus=products&page=1&perpage=30&sort=relevance&term=moo3&type=product

[S53] Merck, Tungsten(VI) oxide. (2023). https://www.sigmaaldrich.com/HK/en/search/wo3?focus=products&page=1&perpage=30&sort=relevance&term=wo3&type=product_name

[S54] Merck, Potassium molybdate. (2023). https://www.sigmaaldrich.com/HK/en/search/k2moo4?focus=products&page=1&perpage=30&sort=relevance&term=k2moo4&type=product

[S55] Merck, Ammonium perrhenate. (2023). https://www.sigmaaldrich.com/HK/en/substance/ammoniumperrhenate2682413598657

[S56] Merck, Selenium. (2023). https://www.sigmaaldrich.com/HK/en/search/se?focus=products&page=1&perpage=30&sort=relevance&term=se&type=product

[S57] F. Scientific, Dibenzyl diselenide, 95%, Thermo Scientific Chemicals. (2023).

[S58] Merck, Manganese(III) acetylacetonate. (2023). https://www.sigmaaldrich.com/HK/en/product/aldrich/m2284

[S59] Merck, Iron(III) acetylacetonate. (2023). https://www.sigmaaldrich.com/HK/en/substance/ironiiiacetylacetonate3531714024181

[S60] Merck, Cobalt(II) acetylacetonate. (2023). https://www.sigmaaldrich.com/HK/en/substance/cobaltiiacetylacetonate2571514024487

[S61] Merck, Nickel(II) acetylacetonate. (2023). https://www.sigmaaldrich.com/HK/en/search/ni(c5h7o2)2?focus=products&page=1&perpage=30&sort=relevance&term=ni%28c5h7o2%292&type=product

[S62] Merck, Copper(II) acetylacetonate. (2023). https://www.sigmaaldrich.com/HK/en/substance/copperiiacetylacetonate2617613395169
